# sRNA and *cis*-antisense sRNA identification in *Staphylococcus aureus* highlights an unusual sRNA gene cluster with one encoding a secreted peptide

**DOI:** 10.1038/s41598-017-04786-3

**Published:** 2017-07-04

**Authors:** Julie Bronsard, Gaetan Pascreau, Mohamed Sassi, Tony Mauro, Yoann Augagneur, Brice Felden

**Affiliations:** Inserm U1230 Biochimie Pharmaceutique, Université de Rennes 1, Rennes, France

## Abstract

The human pathogen *Staphylococcus aureus* expresses a set of transcriptional factors and small RNAs (sRNAs) to adapt to environmental variations. Recent harmonization of staphylococcal sRNA data allowed us to search for novel sRNAs using DETR’PROK, a computational pipeline for identifying sRNA in prokaryotes. We performed RNA-Seq on Newman strain and identified a set of 48 sRNA candidates. To avoid bioinformatic artefacts, we applied a series of cut-offs and tested experimentally each selected intergenic region. This narrowed the field to 24 expressed sRNAs, of which 21 were new and designated with Srn identifiers. Further examination of these loci revealed that one exhibited an unusual condensed sRNA cluster of about 650 nucleotides. We determined the transcriptional start sites within this region and demonstrated the presence of three contiguous sRNA genes (*srn_9342*, *srn_9344* and *srn_9345*) expressed from the positive strand, and two others (*srn_9343* and *srn_9346*) transcribed from the opposite one. Using comparative genomics, we showed that genetic organization of the *srn_9342-9346* locus is specific to Newman and that its expression is growth-phase dependent and subjected to nutrient deprivation and oxidative stress. Finally, we demonstrated that *srn_9343* encodes a secreted peptide that could belong to a novel *S. aureus* toxin-antitoxin system.

## Introduction

*Staphylococcus aureus* is an opportunistic pathogen responsible for a large spectrum of human and animal infections^1^. The bacterium finely modulates gene expression to efficiently adapt its growth and physiology to the local environment. Besides global transcriptional regulators, small RNAs (sRNAs, also cited as regulatory RNAs when a regulatory function was demonstrated) have emerged as key players in a wide range of biological processes, from central metabolism to virulence and antibiotic resistance^2–4^. In *S. aureus*, sRNAs, are typically 50- to 500-nucleotides (nt) long. Some of them regulate mRNA expression and/or stability without the need of Hfq chaperone^5, 6^. sRNAs with regulatory functions were first discovered by Mizuno in *E. coli* ^7^ and then by Novick in *S. aureus* ^8^. They can modulate target mRNA expression by *cis*- or *trans*-acting mechanisms^2^. *Cis*-encoded sRNAs are transcribed from the opposite strand of an mRNA or another sRNA. Accordingly, they display perfect complementarity with their target sequences, although their ability to bind other RNAs cannot be excluded. *Trans*-encoded sRNAs are transcribed apart from their targets, and usually display only partial complementarity with them.

Over the last decade, several studies have focused on the identification of *S. aureus* sRNAs via bioinformatics, next-generation sequencing (NGS), and other experimental approaches^9–21^. This has resulted in the publication of hundreds of RNA sequences, but their functions are mostly unknown. Most of these studies were done using methicillin-resistant strain N315^11, 18^. In others, newly discovered sRNAs were described based on their genomic location in that particular strain^9, 12, 13, 16^. *S. aureus* sRNA identification has been hampered by the lack of naming consensus and the absence of dedicated annotation files. Recently, we collected all of the sequences published so far, proposed a simple sRNA gene identifier (*srn*) to avoid redundancies, and provided annotation files in the Gene File Format (.gff) to the SRD (*Staphylococcus* regulatory RNA Database)^22^. In this study, the sRNA content of four strains was compared, and their predicted locations suggested that sRNA content is strain-dependent. It therefore seems that global analysis devoted to the identification of novel sRNA is still useful for many bacteria, including *S. aureus*. This was recently confirmed by studies conducted on multilocus sequence typing 8 (ST8) strains, with new sRNAs described in USA300 and NCTC8325^15, 21^. Here, we combined bioinformatics and experimental procedures to identify and characterize novel sRNAs expressed by the *S. aureus* strain Newman^23^. This is a methicillin-susceptible clinical isolate often used to study staphylococcal diseases in animal models^23^, and it belongs to the common ST8 clonal lineage. However, Newman has never before been used to discover new staphylococcal sRNAs, and only three previous studies have focused on the role of already identified sRNAs in Newman. The first described the Srn3580_SprA type I toxin-antitoxin system^24^; the second focused on the anti-virulent role of Srn_3610_SprC^25^; and the third highlighted the functional role of Srn3820_SprX1 in Newman pathogenicity^26^.

In this study, we performed deep RNA sequencing on Newman wild-type and its isogenic mutant *Δsrn_3610_sprc*. We combined the RNA-Seq data with the initial sRNA annotation from the SRD^22^, then used the DETR’PROK pipeline^27^ to identify 48 putative novel transcribed intergenic regions (IGRs). Bioinformatic and experimental analysis of the 48 loci led to the certain characterization of more than 20 novel sRNAs expressed from either the core or accessory genome. Further study of these sRNAs along with RACE-mapping of primary transcripts revealed that Newman has an unusual condensed cluster of five sRNAs. These are all located within 650 nucleotides, and their expression is growth-phase dependent and subject to nutrient starvation and oxidative stress. In the cluster, two RNA pairs are expressed as sense/*cis*-antisense sRNA, with one pair presenting toxin-antitoxin module features, since one of the sRNA was demonstrated to encode and express a 33 amino acid long secreted peptide. This work provides evidence for the existence of at least 21 new *S. aureus* sRNAs, and highlights the unprecedented organization of an sRNA cluster of unknown function. Our approach could be extended to wider experimental conditions on the most represented STs to characterize the full repertoire of staphylococcal sRNA (pan-RNome).

## Results

### Bioinformatic screening identifies 48 novel sRNA candidates in *Staphylococcus aureus* Newman

So far, more than 500 sRNAs are compiled under unique Srn identifiers in the SRD^22^. The majority were identified using high-throughput screening, with sixty experimentally confirmed^22^. Our goal here was to search for novel sRNAs by combining RNA-Seq, bioinformatics, and experimental assessments, thus reducing the false discovery rate inherent to this type of approach. We examined *S. aureus* Newman, a methicillin-susceptible clinical isolate^23, 28, 29^ which has never before been used for sRNA discovery. Whereas previous studies were based on the use of various growth conditions^11, 15, 21^, we compared wild-type Newman to an sRNA mutant strain, as sRNAs can modulate the expression of various targets including transcription factors (MgrA, Rot, SarT)^3^. These transcription factors could, in turn, control sRNA expression. Therefore, we used a strain deleted for Srn_3610_SprC sRNA to see whether that deletion allowed the identification of more sRNAs. Srn_3610_SprC is an sRNA that belongs to the SarA regulon and attenuates virulence^25, 30^. Indeed, in the absence of Srn_3610_SprC, *S. aureus* phagocytosis by macrophages increases, allowing for an internal proliferation of the bacterium and its subsequent release and dissemination into the organism. During the post-exponential phase, we extracted total RNA from isogenic and mutant strains, and prepared RNA-Seq libraries. We then used DETR’PROK^27^, a pipeline recently developed to identify sRNAs in prokaryotes. DETR’PROK assembled, in the non-annotated regions, overlapping reads into clusters representing sRNA candidates. Consequently, the workflow was designed and set to preferentially search for RNAs transcribed in an independent manner rather than mRNA leaders (see Methods for the parameters used). To avoid reexamining previously reported sRNAs, we combined the SRD gene annotation for *srns* ^22^ with gene-coding annotations in GFF format that were downloaded from NCBI. Among the six biological replicates (three wild-type Newman and three Newman *Δsrn_3610_sprc* transcriptomes), there were 18 to 27 novel sRNA candidates per replicate, resulting in a total of 48 independent IGRs that were not from mRNA UTRs (Table 1). Of these, 11 were detected by the framework only in the wild-type strain, while six clusters were only in the mutant (File S1). To verify whether these clusters were specific to the knockout or parental strain, or simply due to limitations in the DETR’PROK clustering process, we annotated the 48 IGRs in our GFF annotation file and used the HTSeq/DESeq pipeline^31, 32^. This pipeline count reads within annotation, normalizes data, and calculates differential expression levels between strains. Running this process on Newman and *Δsrn_3610_sprc* revealed that there were no significant (*p* < 0.05) transcript level variations for any of the IGRs. Consequently, it indicated that none of these 48 clusters were under the control of Srn_3610_SprC. This result can be explained by recent work showing this sRNA to be directly repressed by SarA which therefore significantly lower expression of Srn_3610_SprC in wild-type under normal growth condition^30^.

**Table 1:** RNA-Seq identification of 48 transcribed IGRs within the *Staphylococcus aureus* Newman strain.

IGR	Location	Size (nt)	Strand	Number of replicates	HTSeq mean count	FPKM	Repeated sequences
1	108914	267	−	1/6	29	23	Unique
2	109544	539	−	3/6	33	14	Unique
3	115311	297	−	6/6	3603	2320	Multiple (21)
4	138924	274	−	1/6	15	13	Unique
5	363783	407	+	5/6	30	17	Unique
6	363785	304	−	6/6	20	14	Unique
7	493064	305	+	1/6	41	25	Unique
8	787855	483	−	3/6	14	5	Unique
9	819355	324	+	2/6	21	13	Multiple (26)
10	819365	324	−	2/6	13	7	Multiple (26)
11	824790	554	−	6/6	32	11	Multiple (73)
12	824832	450	+	6/6	1442	642	Multiple (71)
13	846032	175	−	1/6	7	10	Unique
14	897930	466	+	5/6	840	348	Unique
15	1005976	363	+	1/6	4	2	Unique
16	1050586	474	−	4/6	5044	2534	Unique
17	1079951	348	−	5/6	2448	1536	Unique
18	1102344	234	+	3/6	15	11	Unique
19	1103623	461	−	2/6	3511	1502	Copy 1
20	1141544	424	+	5/6	110315	55478	Unique
21	1175020	490	−	4/6	294	136	Unique
22	1175024	480	+	4/6	23	9	Unique
23	1211256	286	+	1/6	11	8	Multiple (21)
24	1332002	154	−	1/6	10	14	Unique
25	1344201	624	+	3/6	15	4	Unique
26	1346470	479	+	4/6	29	14	Unique
27	1350072	600	+	3/6	170	57	Unique
28	1419610	342	+	1/6	12	4	Unique
29	1462664	341	+	4/6	86	47	Unique
30	1462677	229	−	2/6	22	18	Unique
31	1471642	205	−	1/6	28	29	Unique
32	1526641	270	−	1/6	10	5	Multiple (4)
33	1764262	147	+	6/6	1	0	Multiple (14)
34	1930943	264	+	1/6	38	20	Unique
35	1963532	241	−	4/6	13	5	Unique
36	1979939	388	+	3/6	102	49	Unique
37	2017461	520	+	5/6	1750	689	Copy 2
38	2018908	364	+	6/6	146395	81475	Unique
39	2018949	1127	−	4/6	557	77	Unique
40	2019372	279	+	6/6	3692	2685	Unique
41	2083121	419	−	3/6	226	92	Unique
42	2345975	119	+	1/6	9	20	Multiple (2)
43	2436048	299	−	6/6	9	6	Multiple (18)
44	2456865	465	−	2/6	13	5	Multiple (14)
45	2548150	280	−	2/6	12	12	Unique
46	2548156	265	+	2/6	684	664	Unique
47	2605426	783	−	5/6	486	98	Unique
48	2839262	171	−	1/6	220	280	Unique

IGR, intergenic region; nt, nucleotides; FPKM, fragments per kilobase of exon per million reads mapped. When a sequence is repeated over the genome (at least 60% of the sequence of the candidate) the number of repetitions appears in parentheses.

### Bioinformatic and manual curation of IGRs narrows the field to 17 sRNA candidates

Previous studies showed that erroneous annotation of repeated sequences or UTRs as sRNAs is likely to occur, and therefore that it may be challenging to identify novel sRNAs^11, 14, 22^. To address this issue, each sequence extracted from DETR’PROK’s output was systematically submitted to BLAST to determine whether the candidate sequence appeared elsewhere in the Newman genome. From the 48 IGRs listed in Table 1, 35 were found to be unique whereas the others were retrieved at multiple locations in Newman (from 2 copies to more than 70). To reduce this set to the most relevant ones (the unrepeated and truly expressed ones), we applied several cutoffs. First, IGRs identified 10 or more times in the Newman genome were discarded to avoid characterization of repeated sequences. Second, we removed all IGRs with a mean HTSeq count (number of paired-end fragments)^31^ lower than 20. Third, FPKM (fragments per kilobase of exon per million reads mapped) normalization^33^ was done to take into account not only the sequencing depths, but also the lengths of the transcribed regions. We eliminated all the clusters with an average FPKM of less than 9. The combination of these cutoffs led to the removal of 23 clusters (File S2). We then continued curation by using the Artemis genome browser^34^. This led to the elimination of 8 additional IGRs associated with UTRs, or misannotated genes that mostly corresponded to IGRs identified in few replicates (File S2). Reducing the number of candidates was essential to limit the spread of false sRNAs and so we could focus on the most accurate ones. We were thus able to make a new list of 17 IGRs (Table 2), 11 of which were located in the Newman accessory genome^23^. Interestingly, two of them (IGR_1103623 and IGR_2017461) located respectively in φNM1 and φNM2 are almost identical, with more than 98% sequence identity over 380 nt (Table 1, copies 1 and 2). Five candidates were located directly downstream or upstream of an already known sRNA. Altogether, our results strongly suggested the presence of additional sRNAs expressed in Newman strain.

**Table 2:** Bioinformatic curation narrows the list of possible new sRNAs found in *Staphylococcus aureus* Newman strain to 17 candidates.

IGR	Location	Core or accessory genome	Gene upstream	Gene downstream	GC %
5	363783	φNM4	srn_3820.2/+	NWMN_0314/−	35.8
6	363785	φNM4	srn_3820.2/+	NWMN_0314/−	38
14	897930	core	NWMN_0810/+	NWMN_0811/+	28.7
16	1050586	core	srn_2330/−	NWMN_0946/+	28.4
17	1079951	core	NWMN_0973/+	NWMN_0974/+	29.5
19	1103623	φNM2	NWMN_0995/+	NWMN_0996/+	30.5
20	1141544	φNM2	Phage lysin amidase/+	isdB/−	42.1
21	1175020	vsaγ	NWMN_1071/−	NWMN_1072/−	23.2
22	1175024	vsaγ	NWMN_1071/−	NWMN_1072/−	23.1
27	1350072	core	NWMN_1224/+	NWMN_1225/+	28.3
36	1979939	φNM1	NWMN_1768/−	srn_9350/+	27.8
37	2017461	φNM1	NWMN_1810/−	NWMN_1811/−	29.6
38	2018908	φNM1	NWMN_1811/−	NWMN_1812/+	38.9
40	2019372	φNM1	NWMN_1811/−	NWMN_1812/+	32.5
41	2083121	φNM3	NWMN_1870/−	srn_3770/+	25.5
47	2605426	core	NWMN_2367/−	NWMN_2368/−	34.8
48	2839262	core	lip/−	hisIE/−	27.3

The locations listed are based on coordinates in the *Staphylococcus aureus* Newman strain. Core and accessory candidates were determined based on the genome sequence published by Baba *et al*.^23^.

### Experimental validation of 17 novel sRNAs expressed from core and accessory genomes

**Figure 1:**
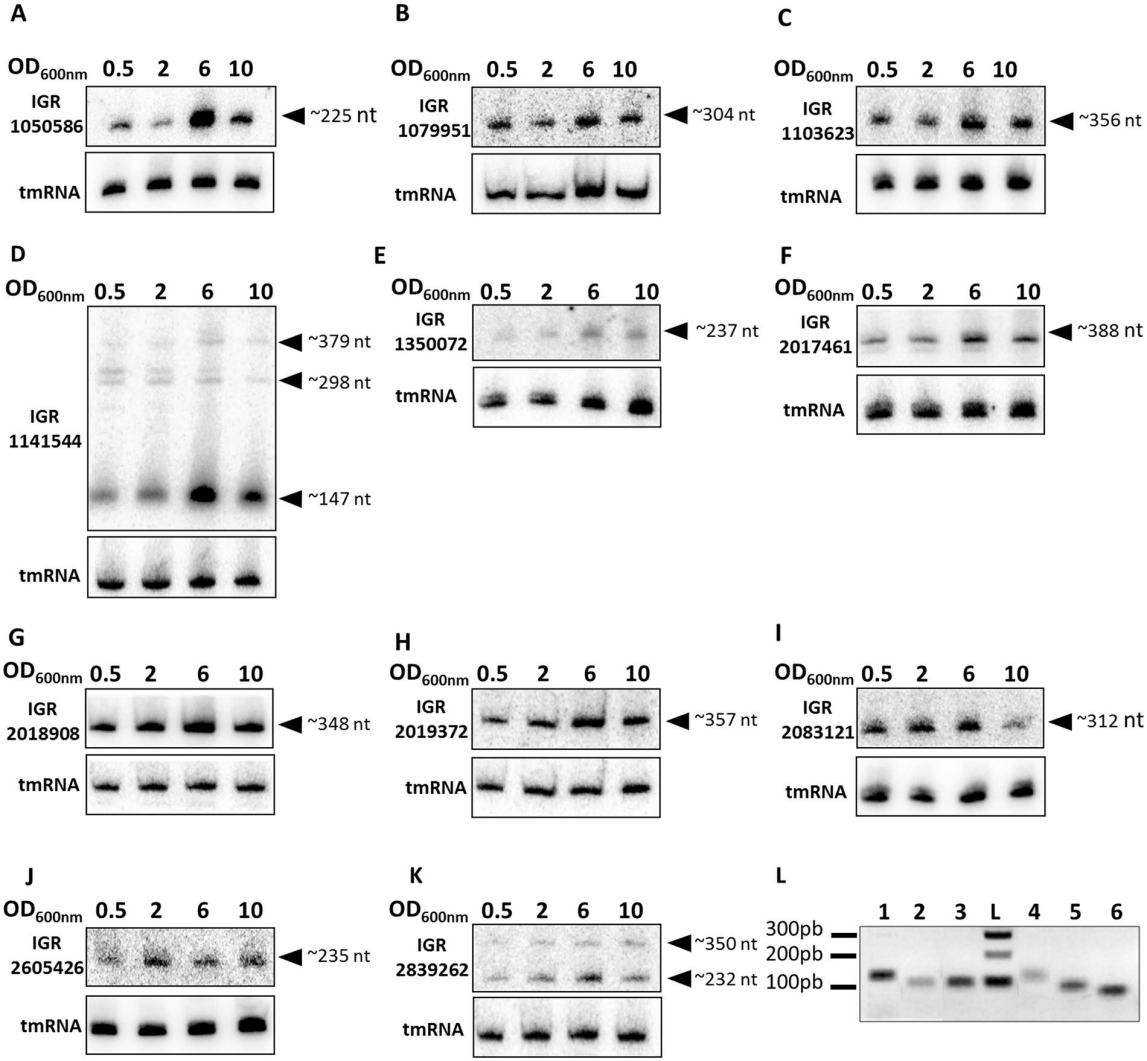
Experimental validation of 17 novel RNAs expressed from *Staphylococcus aureus* Newman strain. Northern blots (**A–K**) were performed on RNA extracted from cells collected at an OD_600nm_ of 0.5, 2, 6, and 10. tmRNA, transfer-messenger RNA used as an internal control; nt, nucleotides. (**L**) RT-PCR was performed on RNA extracted from cells collected at an OD_600nm_ of 6. Lanes 1 to 6 show IGR_ 1979939, IGR_ 363783, IGR_ 363785, IGR_ 1175020, IGR_ 1175024, and IGR_ 897930, from left to right. The data illustrate one representative experiment among three independent biological replicates. The gels presented here were cropped for clarity purpose.

Using RNA-Seq data and after curation, the number of sRNA candidates was reduced considerably, from 48 to 17 IGRs. To avoid false positives, we needed to validate their expression by other means. Transfer-messenger RNA (tmRNA) was used as an internal control for northern blot analysis monitoring the expression of the 17 IGRs during growth in brain-heart infusion (BHI) medium at four time points (Fig. S1). In this way, the expression of 11 sRNAs was confirmed (Fig. 1A–K), corresponding to the regions with the highest HTSeq counts in the RNA-Seq dataset (Table 1). As six of the 17 new sRNAs were not detected by northern blot analysis, RT-PCR was conducted, and this confirmed that these IGRs were in fact transcribed (Fig. 1L). IGR copies 1103623 and 2017461, which share 98% identity, were detected by northern blots using probes directed against the sequence not conserved between the two sRNAs, indicating that they are both transcribed in Newman (Fig. 1C,F). The relative quantification of sRNAs revealed that the transcript levels expressed from IGRs 1050586, 1141544, and 2019372 all varied as a function of the growth phase (Figs 1A,D,H and S2). When compared with the sRNAs extracted in the middle of exponential phase (OD_600nm_ = 0.5), a 2- to 4-fold increase in RNA levels was observed at an OD_600nm_ of 6, followed by a decrease (although for IGR_1141544 this was moderate) (Fig. S2). The transcript length of each RNA was estimated using tmRNA (358 nt) combined with a prestain marker for small RNA (Fig. 1A–K). For the most part, the calculated lengths were close to those estimated by DETR’PROK and/or by visualization using Artemis software. However, significant differences were observed for a few sRNAs. For instance, IGR_1141544 (Fig. 1D) exhibited a strong northern blot signal for an approximately 150-nt long transcript with the presence of higher bands of weaker intensities. Similarly, northern blot revealed two RNA fragments for IGR_2839262 (Fig. 1K). To assess whether the presence of additional bands was due to RNase enzymatic cleavages, contribution of RNase Y and RNase III^35^ to a potential processing of IGR_2839262 was tested and rejected (Fig. S3). This suggests (i) that one or more other RNases (RNase J1 or J2, RNase R, etc.) may process the RNA; (ii) that cleavage may not be RNase-dependent; or (iii) that the multiple bands result from distinct transcriptional units.

To further examine the existence and conservation of these 17 sRNAs, we monitored the expression of the most-expressed transcripts (Fig. 1A–K, Table 1) in several *S. aureus* reference strains. To do that, we ran a BLAST query to look for homologous IGR sequences in the *S. aureus* N315, HG003, USA300, and UAMS1 genomes^36–39^. Among those that northern blots showed as being expressed, only IGRs 1050586, 1079951, 2083121, and 2605426 were retrieved in each of the four genomes, and their transcription was confirmed by northern blots (Fig. S4). Interestingly, IGR_2083121 was reported to belong to the accessory genome (φNM3) of Newman^23^. Taken together, our data confirmed the existence of at least 17 novel sRNAs located either in the core or accessory genome of Newman, including four sRNAs conserved among various *S. aureus* strains.

### Screening for *cis*-antisense transcription specifically targeting the novel sRNAs raised the number of sRNAs to 22

**Figure 2:**
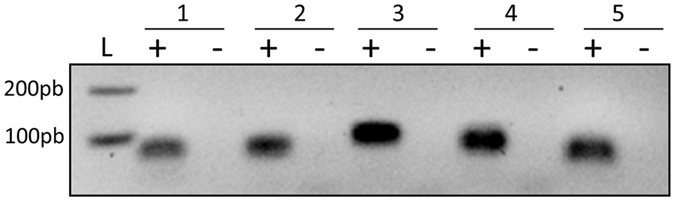
Antisense transcription detected on the opposite strand of some newly identified RNAs through MiSeq RNA sequencing. RT-PCR was performed on RNA extracted from cells collected at an OD_600nm_ of 6. Lanes 1 to 5 show IGR_1080208, IGR_1141547, IGR_1141775IGR, IGR_2019414 and 2019159, from left to right after cDNA synthesis (+) or after DNase treatment (−). The gel presented here was cropped for clarity purpose.

Studies aiming at identifying novel sRNAs have reported antisense transcription of both coding sequences and sRNA genes^14, 40^. Two sense-antisense pairs have been identified^24, 41^ among the sRNAs expressed from *S. aureus* pathogenicity islands^18^. Here, we noted that there are sense-antisense pair characteristics in four of the experimentally confirmed RNAs (identified by RT-PCR in IGRs 363783, 363785, 1175020, and 1175024). Two by two, they share the same loci but are transcribed from opposite strands. This made us wonder whether the remaining 13 transcripts were associated with transcription located on the opposite strand and undetected by DETR’PROK due to the average library size and the sequencing mode (2 × 100 bp). To investigate this, and because so far the *S. aureus* RNAs transcribed from the opposite strand of a known sRNA are usually smaller than 100 nt^14, 22^, we performed a new RNA-Seq. In this one, we made and purified cDNAs with a minimal length of about 70 bp and generated single reads of 50 nt (sequenced using MiSeq), while the size of the smallest fragments generated for HiSeq sequencing was larger than 100 bp. We then used Artemis to specifically look for reads mapping on the opposite strand of these 13 RNAs, finding five additional read clusters (Table 3). Interestingly, we identified two of these (named as2 and as3) on the opposite strand of sRNAs expressed from IGR_1141544. Probes and primers were designed to verify whether transcripts could be detected by northern blots and RT-PCR. Even though no bands could be observed by northern blot using various DNA probes, all transcripts were individually recovered by RT-PCR (using specific primers for reverse transcription). This suggested low expression levels of these RNAs under our experimental conditions (Fig. 2). Altogether, this increased the novel sRNA count to 22, and highlights the importance of combining several sequencing protocols and detection methods for thorough and exhaustive sRNA identification.

**Table 3:** Transcription detected on the opposite strand of the novel RNA transcripts determined by RNA-Seq.

RNAs	Coordinates*	Targeted IGR	Strand	Length
as1	1080216–1080299	1079951	+	83
as2	1141494–1141607	1141544	−	113
as3	1141665–1141817	1141544	−	152
as4	2019015–2019244	2018908	−	229
as5	2019390–2019490	2019372	−	100

*The coordinates were determined using Artemis.

### Newman strain contains an unusual condensed sRNA cluster composed of five transcriptional start sites

**Figure 3:**
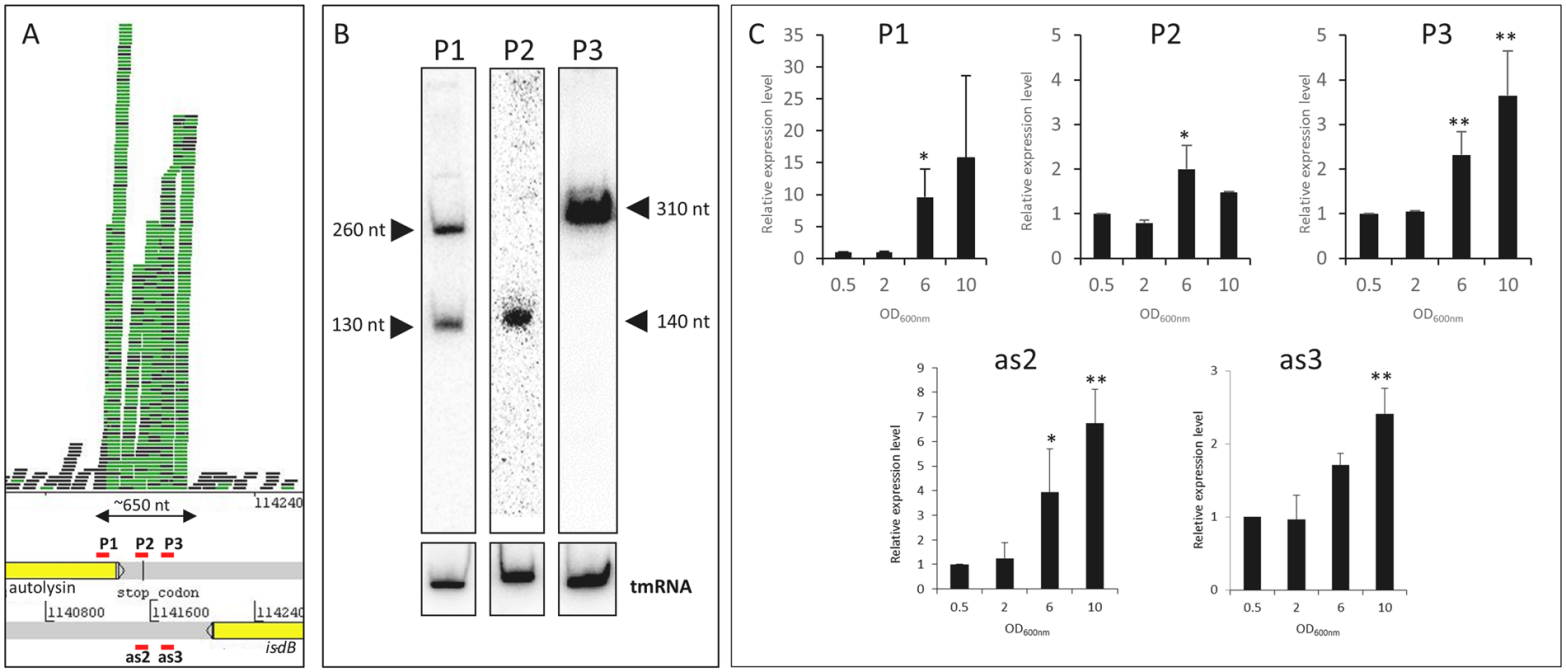
Characterization of an sRNA transcription hotspot in *Staphylococcus aureus* Newman. (**A**) RNA mapping profile of IGR_11415444 visualized with Artemis. P1, P2, P3, as2, and as3 correspond to the position of probes and/or primers for northern blot and RT-qPCR experiments. (**B**) Identification of multiple transcripts expressed from the positive strand of the Newman genome. Total RNA were extracted from cells collected at an OD_600nm_ of 6 and tmRNA was used as an internal loading control. (**C**) Relative expression levels of the IGR_1141544 locus as a function of growth phase. The relative cDNA level was determined using HU as an internal control and OD_600nm_ of 0.5 as a calibrator. The data shown are the means of three independent experiments. A student t-test was performed to determine differences with condition at OD_600nm_ of 0.5 (*p < 0.05, **p < 0.01, ***p < 0.001). The gel presented here was cropped for clarity purpose.

In IGR_1141544, comparison of the transcript lengths determined by northern blots with the sRNA lengths either inferred from DETR’PROK or after Artemis visualization of single ends revealed major differences. While DETR’PROK predicted a single transcript of 424 nt (Table 1) and Artemis suggested a 650-nt one (Fig. 3A), we identified an sRNA about 150-nt long by northern blot, with increased expression levels during growth (Figs S1 and S2). Additional focus using an RNAseY mutant did not evidenced any maturation of a longer transcript (Fig. S3). To try to explain the differences between the predictions and our experimental results, we further investigated this particular sRNA-rich locus located within φNM2. Thorough inspection of RNA-Seq reads specifically mapping this region revealed that transcription of this RNA should start within the CDS 3′ end of NWMN_1039-1, which encodes the φNM2 autolysin amidase domain (Fig. 3A). Based on the heterogeneous depth and on the location of paired-end reads, read mapping profiles suggested the presence of several transcripts (Fig. S5). We therefore designed probes for northern blots targeting different loci (Fig. 3A, P1 to P3). This revealed the presence of several RNAs transcribed from the positive strand in this nearly 700-nt region. Interestingly, northern blot conducted with probe P1 resulted in the detection of two bands of around 130 and 260 nt (Fig. 3B). This suggested the presence of either two distinct transcription start sites (TSSs) or a single TSS with two transcription terminators. Sequence analysis using ARNold^42^ supported the second hypothesis, revealing two Rho-independent terminators: one corresponding to the NWMN_1039-1 3′ end, and another located further downstream. Conversely, probes P2 and P3 identified single transcripts of around 140 and 310 nt (Fig. 3B). Through RT-qPCR, we showed that levels of all three RNA (P1 to P3) were increased at OD_600nm_ 6 and 10 (Fig. 3C), confirming the previously obtained northern blot results (Figs 1 and S2). Likewise, RT-qPCR conducted to monitor RNA levels of as2 and as3 (the MiSeq-identified RNAs; see Table 3) revealed a similar expression profile (Fig. 3C). This indicated that the entire locus responds to changes of growth phase and maybe also to nutrient starvation or cell density. Additionally, the differences observed in the CTs obtained by RT-qPCR confirmed that as2 and as3 were weakly expressed compared to the RNAs transcribed from the positive strand. We then used RACE-mapping on both strands to identify the 5′ and 3′ ends of the different RNA molecules expressed from this tiny region (Table 4). This confirmed the presence of the four transcripts detected by northern blot (Fig. 3B). Furthermore, it proved that the two transcripts detected with P1 share the same 5′ end, while the two 3′ends correspond to the position of predicted Rho-independent terminators and should therefore be considered as a single transcription unit. Because this genomic locus has a very compacted structure, we checked to see whether these novel RNAs were transcribed from distinct TSSs or were the result of the maturation of a long transcript. From total RNA, we degraded mono-phosphorylated RNAs (RNAs issued from post-transcription 5′ processing) using Terminator 5′-Phosphate-Dependent Exonuclease (TEX) followed by polyphosphatase treatment, to enrich the ligation of the TSS RNA. This confirmed that the three positive-strand RNAs are independent transcription units (Table 4). On the opposite strand, RACE-mapping similarly confirmed the presence of two RNAs of 138 and 170 nt, as2 and as3 (Table 4), as found in the previous MiSeq experiments (see above). Therefore, among the 22 previously identified sRNA, we were able to prove the existence of three sRNAs (instead of one) expressed from the positive strand of IGR_1141544, giving us a new total of 24 sRNAs expressed in Newman.

**Table 4:** RACE-mapping characterization of sRNAs transcribed from IGR_1141544.

Target or probe	Coordinates	Strand	Length
P1	1141253–1141502	+	249
P1	1141253–1141398	+	145
P2	1141525–1141656	+	131
P3	1141676–1141990	+	314
as2	1141515–1141653	−	138
as3	1141677–1141847	−	170

### Extensive sRNA end-mapping and assignment of *srn* identifiers

We previously compiled staphylococcal sRNAs into the SRD database under simple *srn* identifiers to harmonize the sRNA repertoire^22^. To generate names for these 24 newly discovered sRNAs, we first determined the 5′ and 3′ ends of the transcripts by RACE-mapping (Table 5). Then, we provided an *srn* identifier for each RNA (Table 5) based on its location on the genome of five reference strains (N315, Newman, JDK6008, NCTC8325, and USA300) as provided in SRD^22^. The nucleotide sequences defined by RACE confirmed all of the previously estimated lengths, and provided additional proof that the two sRNA copies, hereby renamed Srn_9650 (IGR_1103623) and Srn_9660 (IGR_2017461), were transcribed. We also used TEX followed by polyphosphatase treatment to identify a TSS for *srn_3765* (IGR_2083121) at position 2083472 (a 276-nt transcript), while an RNA fragment of only 204 nt was detected without TEX treatment, suggesting maturation of that particular RNA at position 72 (not detected by northern blot, Fig. 1I). TEX-RACE-mapping of Srn_5075 (IGR 2839262) only revealed a 220-nt fragment, indicating that the upper band detected by northern blot is non-specific (Figs 1K and S3). Using the accurate *srn* coordinates, we compared the 24 new sRNA sequences with sRNAs recently identified in USA300 and HG001^15, 21^ and for which the *srn* annotation was not yet available. This showed that, among the 24, only three of them (*srn_2335*, *srn_9345*, and *srn_4635*) were already identified as transcribed in those two recent studies (Table 5). All the 24 transcripts identified in this report fit in the sRNA class, as the longest is shorter than 400 nt. As studies on sRNAs showed that some can contain ORFs^2, 11, 14, 21, 22^, we used ORFfinder to search for their presence within each mapped sequence. This unveiled that 13 sRNAs contained a small ORF (Table 5). We then used them to BLASTP to assess whether they were automatically annotated as potential CDS within *S. aureus* taxon. This revealed that, among the 13 sRNAs that contained an ORF, 10 were predicted to encode a hypothetical small protein or peptide in at least one *S. aureus* strain although transcription of these putative genes (and therefore translation) was not demonstrated. We also used the Uniprot database to search for sequence homology with known proteins or domains. Eight of them did not match any referenced proteins or peptides, while the two remaining shared partial homologies with larger proteins. Together, these data indicate that 14 of the identified transcripts identified are *bona fide* non-coding sRNAs, and that the features of the remaining 10, make them good candidates for sRNA with potential dual-functions. Overall, it shows that the *S. aureus* sRNA content has not been fully uncovered yet.

**Table 5:** Coordinates of 24 novel transcripts determined by RACE-mapping in *Staphylococcus aureus* Newman and their assigned Srn identifiers.

Identifiers	Other names	Genomic coordinates	Strand	Length	ORF	Potential CDS	Homologies with known proteins or peptides
*srn_0795*	*IGR_363783*	363827–364198^a^	+	371	YES	YES	Partial with lipase
*srn_9640*	*IGR_363785*	363785–364009	−	265	NO	NO	None
*srn_2058*	*IGR_897930*	897979–898218	+	239	YES	NO	None
*srn_2335*	*IGR_1050586; tsr18* ^b^	1050865–1051075	−	210	YES	YES	Partial with SufA
*srn_2347*	*IGR_1079951*	1079989–1080300	−	311	YES	YES	None
*srn_2348*	*as1*	1080214–1080281	+	67	NO	NO	None
*srn_9650*	*IGR_1103623*	1103685–1104067	−	382	YES	YES	None
*srn_9342*	*IGR_1141544; P1* ^c^	1141253–1141502	+	249	NO	NO	None
*srn_9343*	*as2* ^c^	1141515–1141653	−	138	YES	YES	None
*srn_9344*	*IGR_1141544; P2* ^c^	1141525–1141656	+	131	NO	NO	None
*srn_9345*	*IGR_1141544; S808* ^d^, *P3* ^c^	1141676–1141990	+	314	NO	NO	None
*srn_9346*	*as3* ^c^	1141677–1141847	−	170	NO	NO	None
*srn_2467*	*IGR_1175020*	1175201–1175405^a^	−	204	NO	NO	None
*srn_2468*	*IGR_1175024*	1175217–1175422	+	205	NO	NO	None
*srn_9348*	*IGR_1350072*	1350145–1350345	+	200	YES	YES	None
*srn_9349*	*IGR_1979939*	1980012–1980322	+	310	YES	NO	None
*srn_9660*	*IGR_2017461*	2017588–2017972	+	384	YES	YES	None
*srn_9670*	*IGR_2018908*	2018982–2019366	+	384	YES	NO	None
*srn_9671*	*as4*	2019068–2019159	−	91	NO	NO	None
*srn_9680*	*IGR_2019372*	2019373–2019729	+	356	YES	YES	None
*srn_9681*	*as5*	2019391–2019458	−	67	NO	NO	None
*srn_3765*	*IGR_2083121*	2083196–2083472	−	276	YES	YES	None
*srn_4635*	*IGR_2605426; S1077* ^d^	2605636–2605853	−	217	YES	YES	None
*srn_5075*	*IGR_2839262*	2839213–2839434	−	221	NO	NO	None

^a^not mapped by RACE; ^b^described by Carroll *et al*.^21^; ^c^described in Fig. 3, Table 3, and Table 4; ^d^described by Mader *et al*.^15^. ORF of 30 codons or more were identified using ORFinder. Potential CDS were searched using BLAST within the non-redundant database. Homologies with known proteins were searched using Uniprot database. Homologies were described as partial when the coverage and length of the amino acid sequence did not exceed 60% of the homologous protein sequence.

### Conservation of newly discovered sRNAs in Sta*phylococcus aureus*

**Figure 4:**
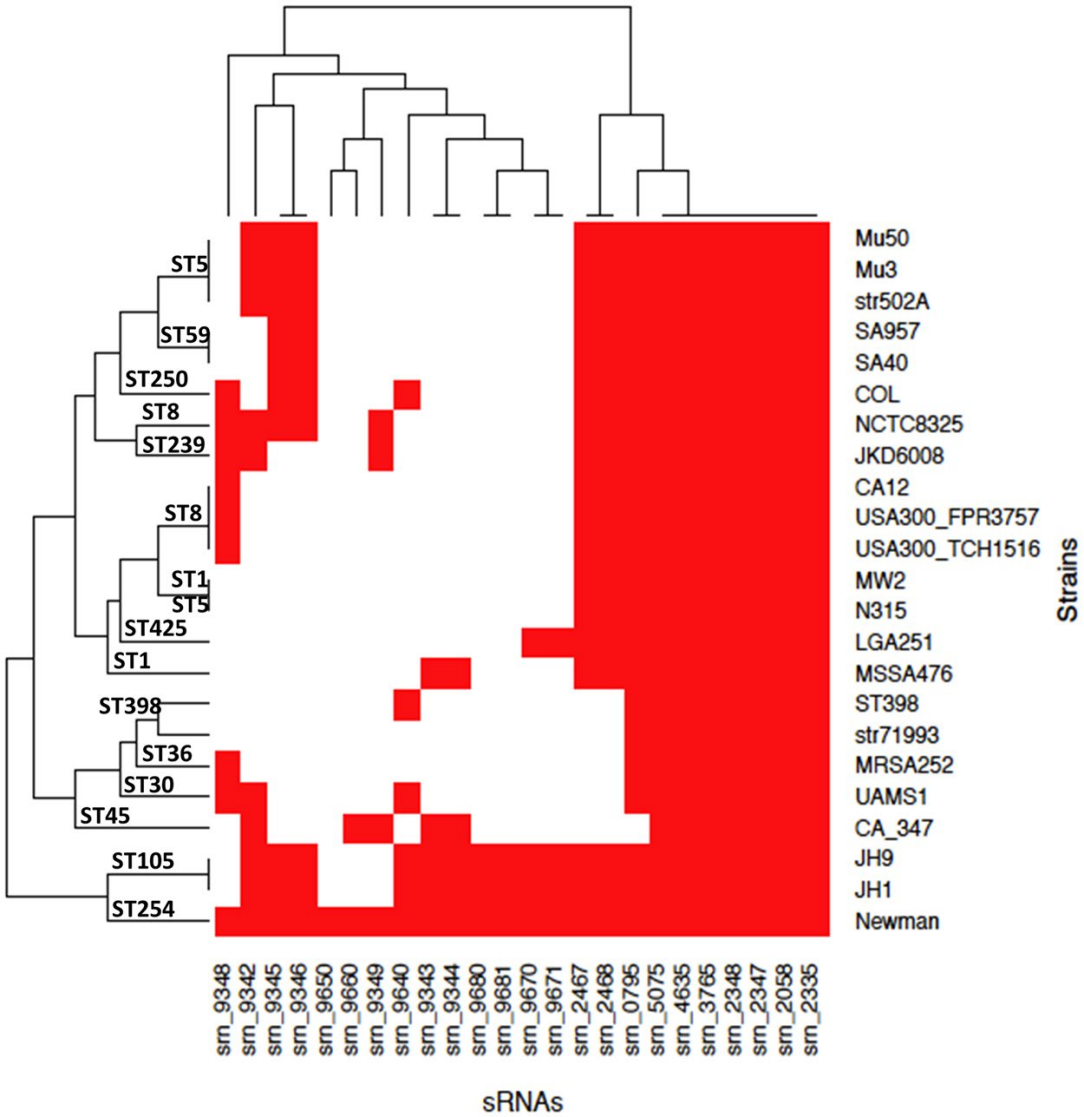
Comparative analysis of the presence and absence of novel *srns* in *Staphylococcus* strains. A heat map was generated based on the presence (red) and absence (white) of *srns* in strains belonging to various clonal complexes. The data were clustered based on *srns* and genome sequence identities.

The assignment of *srn* identifiers followed with their alignment showed that some sRNAs (mostly those starting with srn_9XXX) were not detected in the four SRD reference strains (N315, NCTC8325, JKD6008, and USA300). Therefore, to estimate the conservation levels of the 24 novel sRNAs, we aligned their sequences to assess their presence and absence in 22 *S. aureus* strains that belong to well-characterized clonal complexes (Fig. 4). This revealed that 7 of them (*srn_2058*, *srn_2335*, *srn_2347*, *srn_2348*, *srn_3765*, *srn_4635*, and *srn_5075*) are conserved among several *S. aureus* strains and have high nucleotide sequence identities (Fig. S6). The analysis of the presence and absence of the 24 sRNAs showed similar sRNA content in Newman, JH1, and JH9. However, Newman is the sole strain that has both copies *srn_9650* and *srn_9660* within its genome, with *srn_9650* unique to Newman and *srn_9660* also encoded in CA-347, an MRSA USA600 strain isolated in 2005 from bloodstream infection. Interestingly, only JH1 and JH9 have the five sRNAs identified in Newman *srn_9342*-*9346* cluster (IGR_1141544), while other strains contain none or just two or three of these sRNA genes (Fig. 4). MSSA476 contains the *srn_9343*/*srn_9344* genes, but *srn_9342* is absent for the first 156 nt and located downstream of another autolysin amidase domain. Collectively, these results show that some of the *srn* genes we identified here are well-conserved within *S. aureus*, while others are strain- or lineage-specific.

### Genetic organization of the *srn_9342-9346* locus is unique to Newman strain

**Figure 5:**
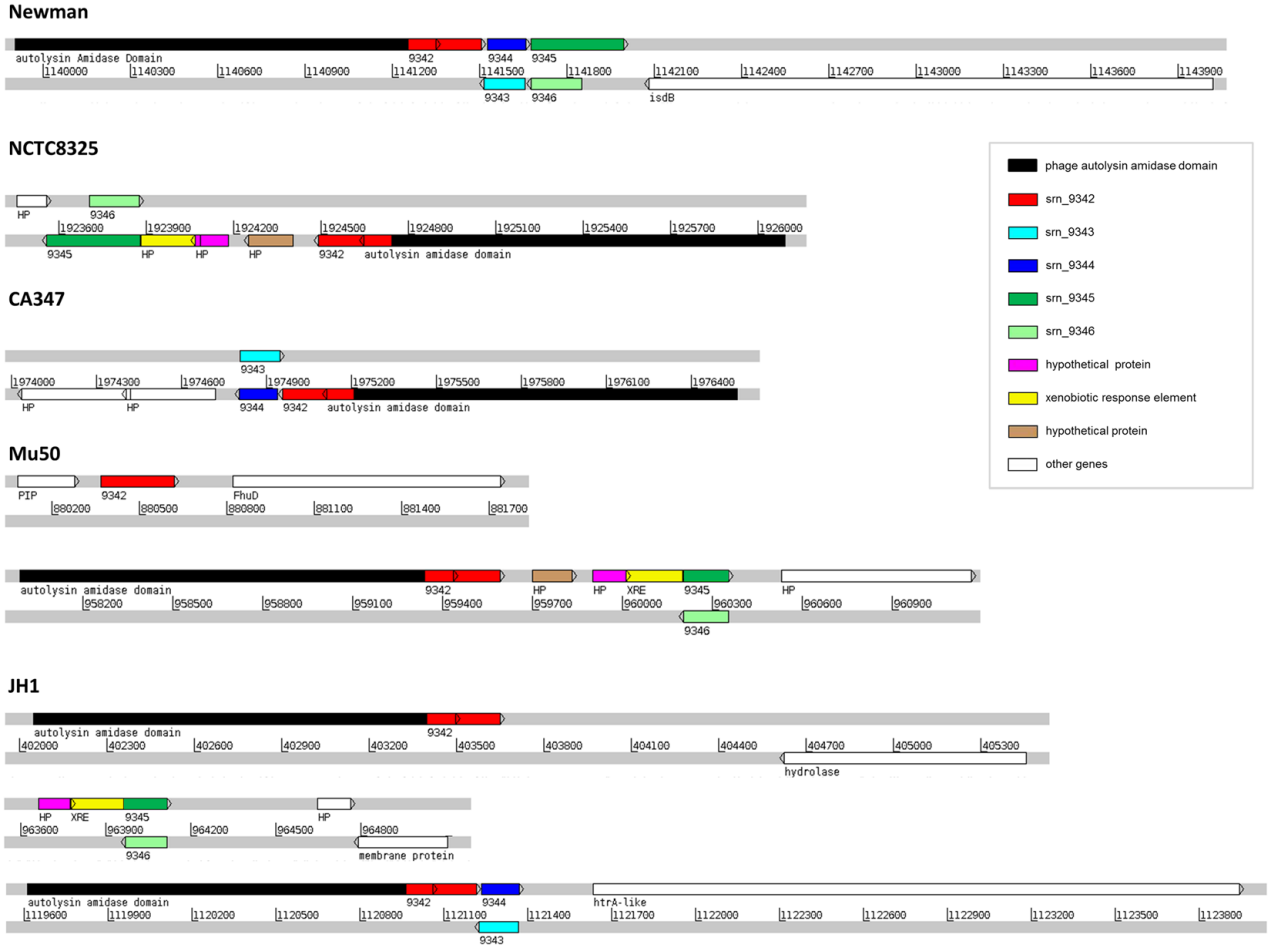
Comparative genomics of the genetic organization of the *srn_9342–9346* cluster. The genetic organization of the *srn9342*–*9346* locus from *Staphylococcus aureus* Newman strain was compared with that of the NCTC8325, CA347, Mu50, and JH1 strains.

The analysis of these new sRNAs in various *S. aureus* genomes showed significant differences in the distribution of the five sRNAs expressed from the *srn_9342-9346* locus. Therefore, we compared the genetic organization of this *srn_9342-9346* locus in Newman, NCTC8325, CA-347, Mu50, and JH1 strains, all from different lineages (Fig. 4) and representative of the genomic distribution in *S. aureus* of these five sRNAs in *S. aureus*. This revealed that Newman is the only strain that has this highly compacted *srn* gene cluster (Fig. 5). In Newman, the *srn* set is located at the 3′ end of pathogenicity island φNM2, and between an autolysin amidase domain and *isdB*, a gene encoding a hemoglobin receptor required for heme iron utilization^43^. NCTC8325 is characterized by the presence of *srn_9342* (also located at the 3′end of an autolysin amidase domain) and *srn_9345/srn_9346*, whereas a three-CDS insertion occurred where Newman has *srn_9343*/*srn_9344*. In CA-347, *srn_9342*-*srn_9344* organization is conserved, but the last two sRNA genes are lacking. Interestingly, Mu50 and JH1 have two copies of *srn_9342* (Fig. 5). In Mu50, the first copy is retrieved as a single *srn* upstream of *fhuD* (and therefore dissociated from an autolysin), and an organization similar to that of NCTC8325 is also present elsewhere in the genome. In JH1, three loci contain sRNA genes of Newman’s *srn_9342-srn_9346* locus, with *srn_9342* again found in two copies (the first at the 3′ end of an autolysin amidase gene). The *srn_9345/srn_9346* gene cluster displays an organization that resembles that of Mu50. In a third genomic environment, *srn_9342* and *srn_9343*/*srn_9344* are located next to another autolysin amidase domain, upstream from an HtrA-like protein coding-gene. Therefore, the comparative analysis showed that the genetic organization of the accessory *srn_9342-9346* sRNA genes is specific to Newman, and that differences in the nearby genetic environment may induce differences in sRNA expression patterns.

### Srn_9343 encodes a peptide secreted in the extracellular medium

**Figure 6:**
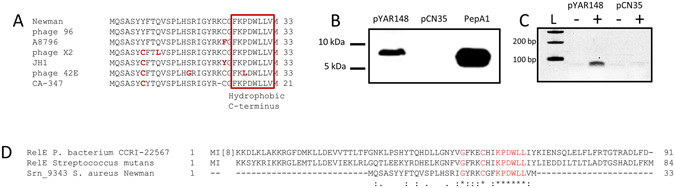
*srn_9343* encodes a peptide expressed in *Staphylococcus aureus* that is secreted extracellularly. (**A**) Sequence alignment of the Newman peptide sequence with the *S. aureus* annotated peptides. (**B**) Western blot analysis of Srn_9343 flagged-peptide expression from pYAR148 (Table 6) under the *pVeg* promoter. This is compared to empty vector (pCN35) and to PepA1 from Sayed *et al*.^66^. (**C**) RT-PCR analysis of the expression of *srn_9343* under the P*veg* promoter. (**D**) Sequence alignment of Srn_9343 with RelE toxins. The experimental data shown here are representative of three independent experiments. The dot and gel presented here were cropped for clarity purpose.

Analysis of the distribution of *srn_9343/srn_9344* revealed that their presence is less conserved than that of the other genes in the locus. While no ORF was identified on the positive strand of the *srn_9342-9346* locus, analysis of the minus strand revealed a small ORF within the *srn_9343* sequence (Table 5). It contains a start codon located downstream of a putative RBS and therefore potentially code for a 33-amino acid peptide. This putative peptide sequence was predicted and annotated as hypothetical protein in NCBI database only in three *S. aureus* strains and three phages (Fig. 6A), with a predicted molecular weight of 3.9 kDa. To assess whether it is produced by *S. aureus*, we cloned *srn_9343* under the control of its own promoter by including up to 250 nt upstream in translational fusion with a 3xFLAG (adding a 2.9-kDa epitope at its C-terminus). Western blot analysis did not reveal any translation of the fusion peptide in cell extracts or in the Newman supernatant (Fig. S7), indicating a potential down-regulation of its expression under these conditions. Therefore, to avoid any regulatory effects due to *srn_9342-9346* locus expression, we used a P*veg* constitutive promoter^44^ and transformed N315, chosen because its genome includes none of these five *srns* (Fig. 4). Although Srn_9343 peptide expression was not detected in cell extracts, it was seen in the N315 supernatant after culture in BHI broth (Fig. 6B), indicating that the peptide is produced at the expected size (Fig. 6C) and secreted. Analysis of the amino acid sequence revealed that it does not share significant similarities with phenol-soluble modulin^45, 46^, but presents a hydrophobic C-terminus. This C-terminus domain is similar to a RelE addiction-module toxin domain in *Streptococcus mutans* (E-value 0.004) and *Peptostreptococcaceae bacterium* (E-value 5. 10^−5^). Altogether, the presence of transcription on both strands, as for Srn_3580_SprA^24^, and similarity with the RelE toxin suggests that *srn_9343/9344* could belong to a putative toxin-antitoxin system.

### Expression of novel *S. aureus* Srns under various stress conditions

**Figure 7:**
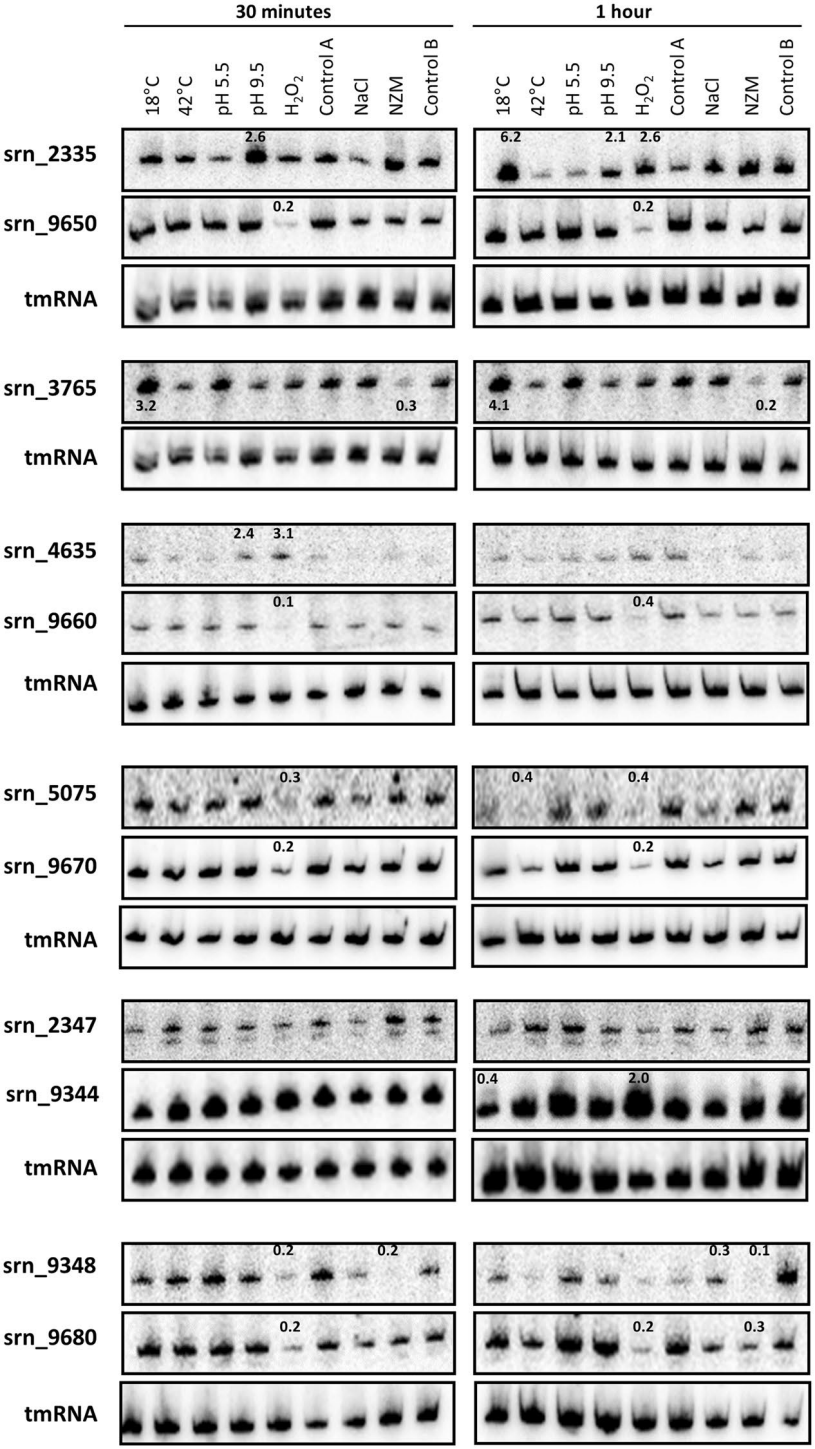
Expression of *srns* monitored under various stress. *Staphylococcus aureus* Newman strain was cultured in TSB broth to an OD_600nm_ of 2 (exponential growth phase). Stress was induced by the addition of H_2_O_2_, HCl, or NaOH, or by changing the temperature to 18 °C or 42 °C. Control A corresponds to cells maintained in TSB under normal conditions. In parallel, other cultures were centrifuged at 4500 rpm for 8 min at RT and pellets resuspended in TSB supplemented with 1 M NaCl (mimicking osmotic stress), NZM medium (emulating a stringent response), or fresh TSB (control B). The relative expression levels are indicated where appropriate, and were calculated after quantification of the dots using control A or B as calibrators. For all experiments, tmRNA was used as an internal loading control. The gels presented here were cropped for clarity purpose.

Many sRNAs respond to specific environmental modifications and help regulate dedicated physiological networks^12, 13, 47, 48^. Therefore, the expression of the 11 *srns* detected by northern blot was assessed under various conditions, including some that mimic the changes encountered by *S. aureus* during host infection. Cells were grown in TSB until reaching the exponential phase of growth (OD_600nm_ = 2), then subjected to stresses related to pH (acidification and alkalization), temperature (heat and cold shocks), oxidation, osmotic levels, and nutrient availability. After either 30 or 60 minutes of stress, the sRNA levels were monitored by northern blot and compared with those produced under unstressed conditions (Fig. 7). Among all of the tested transcripts, Srn_2347 was the only sRNA which had an unwavering transcript level no matter the stress applied. After 30 minutes of oxidative stress, the RNA levels decreased 3- to 10-fold in six Srns (Srn_5075, 9348, 9650, 9660, 9670, and 9680, a group which includes the two sRNA copies). This was also observed after one hour in all of them, except Srn_9348 (Fig. 7). Conversely, Srn_4635 was temporarily overexpressed under oxidative stress (about 3-fold after 30 minutes), while Srn_2335 and Srn_9344 were both overexpressed after one hour in the presence of 10 mM H_2_O_2_. Likewise, Srn_2335 and 4635 transcript levels increased by more than 2-fold after 30 minutes in alkaline conditions (pH 9.5), and remained high in Srn_2335 after one hour of stress. Nutrient deprivation (NZM) in both Srn_3765 and Srn_9348 led to decreased RNA levels after both 30 minutes and 1 hour. Similarly, Srn_9680 levels were around 3-fold lower after one hour in NZM medium. Cold shock (18 °C) in Srn_3765 led to increased RNA levels (3- and 4-fold after 30 minutes and one hour, respectively), whereas Srn_2335 level was multiplied by six after 1 hour. Finally, heat shock (42 °C) led only to an induction of Srn_5075. Interestingly, the two copies (Srn_9650 and Srn_9660) as well as Srn_9670 and Srn_9680 (located near Srn_9660) respond to the same stresses, suggesting similar regulatory mechanisms.

**Figure 8:**
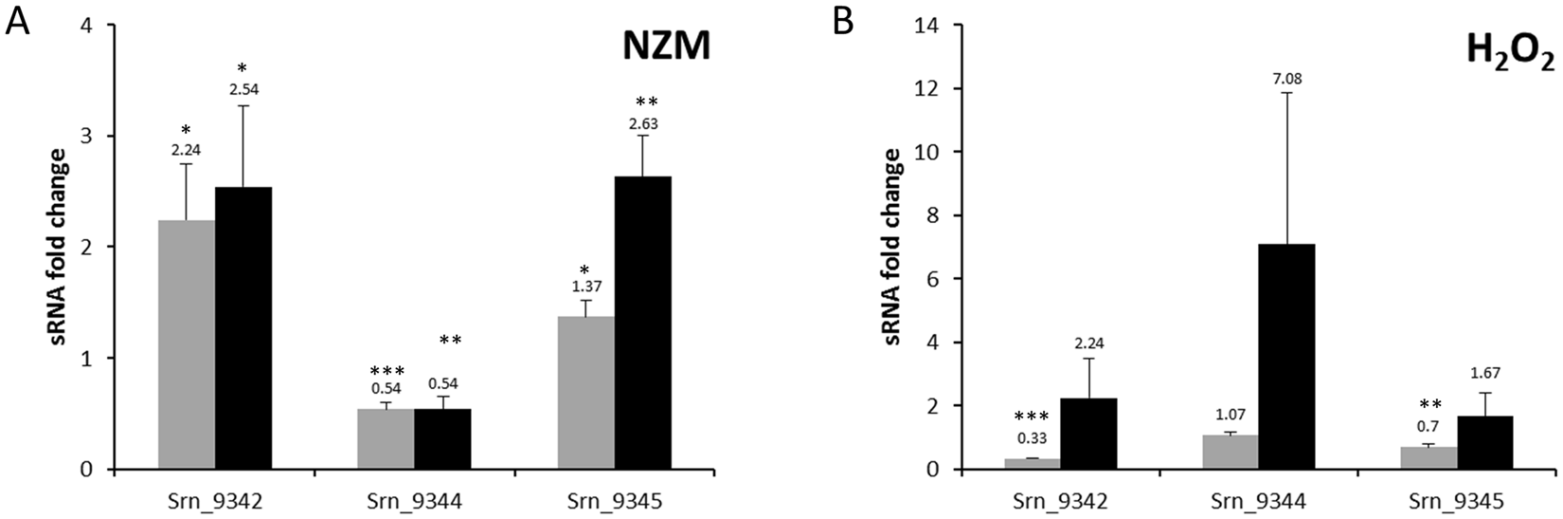
Differential expression of the *srn_9342-9346* locus. sRNA expression levels (**A**) after nutrient starvation in NZM medium and (**B**) under oxidative stress (10 mM H_2_O_2_). The fold changes were calculated after 30 minutes (grey bars) and 1 hour (black bars) of stress using the TSB condition as a control. HU (for NZM) or 5 S cDNA levels were used as internal controls. Data presented are the mean of three independent experiments, all performed in duplicate. A student t-test was performed to determine differences with unstressed condition (*p < 0.05, **p < 0.01, ***p < 0.001).

Going further, to verify whether the whole *srn_9342-9346* locus responds to stress similarly to *srn_9344* (which is sensitive to oxidative stress and cold shock), we investigated the expression levels of these *srns*. We used RT-qPCR rather than northern blot as we found that Srn_9343 and Srn_9346 are expressed at low levels in RNA extracts (Fig. 2). After H_2_O_2_ exposure and nutrient starvation, data analysis revealed differences in the sRNA levels (Fig. 8). In NZM, Srn_9342 RNA levels doubled after 30 minutes and 1 hour, while Srn_9345 induction was observed later, after 1 hour of stress (Fig. 8A). Conversely, Srn_9344 RNA levels decreased slightly. Under oxidative pressure, Srn_9342 RNA levels dropped by around three-fold after 30 minutes, then recovered after 1 hour (Fig. 8B). The levels of the other sRNAs remained nearly identical, although Srn_9344 increased by 7-fold after 1 hour. Together, these data show that transcript levels of 15 novel Srns vary under specific stresses (see summary in File S3). This suggests that their expression is tightly regulated and that they must be involved in distinct physiological pathways. In addition, RT-qPCR analysis of differential expression of the *srn_9342-9346* locus (Fig. 8) showed that the RNA levels of its components can be influenced by nutrient deprivation and oxidative stress.

## Discussion

The last decade has led to the discovery of most *S. aureus* sRNAs, although their functions remain unknown in most cases^22^. This exponential rise in the identification of sRNAs expressed by this major pathogen was enabled by the wide availability and affordability of next-generation sequencing technologies. So far, studies devoted to the detection of novel sRNAs have focused on a few strains, and the results were recently compiled into a dedicated database^22^. Additionally, GenBank files that include sRNAs are available for three *S. aureus* strains^21^. It was necessary to clarify the count of already published sRNAs, so simple identifiers were provided for all of them in addition to their gene coordinates. This mandatory step allows for RNA-Seq studies of the whole transcriptome (mRNA, tRNA, rRNA and sRNA) and facilitates the identification of new sRNAs. Recent publications have demonstrated that knowledge of the full repertoire, the pan-sRNome, has not yet been achieved^15, 21^, as it varies significantly according to the strain^22^. *S. aureus* epidemiology indicates that clonal complex 8 lineages are widely distributed worldwide and dominate human carriage and infections^49^. We therefore continued our efforts to extend the *S. aureus* pan-sRNome by studying the Newman clonal complex 8 strain. This strain has been fully sequenced^23^ and used successfully in animal models of staphylococcal infections, but its sRNA content is quite under-investigated.

We began by using the DETR’PROK framework^27^ to search for unannotated transcribed regions within the Newman IGRs. This resulted in the identification of 48 loci for which RNA-Seq data allowed read clustering. Although DETR’PROK identified sRNA content differences between the Newman and the Newman *Δsrn_3610_sprc* strains, differential expression analysis revealed no statistical differences between the two. Previous studies underlined the high probability of identifying repeated sequences, or 5′ or 3′ UTRs^11, 14, 22^. To avoid this issue, we assigned specific parameters to DETR’PROK, used several cut-offs, systematically used BLAST, and viewed the read mappings to determine whether sRNA candidates seemed to be independent transcriptional units. In addition, misannotated genes were removed during a final manual cull (see File S2) to avoid the identification of putative artefacts. Although we cannot exclude the removal of some true sRNAs, by being safe the combination of these measures narrowed down the candidate set to 17 IGRs, and these were subsequently confirmed by northern blot and/or RT-PCR. None of them were previously reported in the SRD^22^ or BSRD databases^50^ devoted respectively to *Staphylococcus* and bacterial sRNA.

A careful study of the 17 transcribed IGRs began with MiSeq, which led to the discovery of five additional sRNAs. This was completed with RACE-mapping, which characterized the 24 independent transcriptional units or primary transcripts. According to Rfam and Riboswitch finder^51, 52^, the 24 novel sRNAs do not present the sequence and structural characteristics of riboswitches. An in-depth study of IGR_1141544 revealed the presence of three TSSs on the plus strand, and two sRNAs transcribed from the minus strand at the same locus as Srn_9344 and Srn_9345. Such transcripts are common in bacteria with the discovery of natural antisense transcription^40, 53, 54^. However, we found that this locus’ organization is specific to the Newman strain and unusual for sRNAs, and on the positive strand the transcription starts within the 3′ region of an autolysin amidase CDS in the 3′ region. Interestingly, no ORFs were found on the positive strand along this approximately 700-nt transcribed region. RACE-mapping of Srn_9342 surprisingly indicated that two terminators are used, generating two distinct 3′ ends. Its transcription starts within the autolysin coding sequence, mostly leaked through a first rho-independent terminator (which is from autolysin), and when it reaches the next rho-independent terminator the transcription stops. Analysis of the minus strand revealed the presence of an ORF within *srn_9343* along with an RBS upstream the predicted AUG initiation codon. Using 3xFLAG, we showed that the Srn_9343 peptide is produced and secreted. However, failure to express this peptide in Newman suggests the existence of a specific regulatory mechanism that shuts down *srn_9343* expression and translation. Looking outside the *Staphylococcus* genus, this peptide sequence presents similarities to the bacterial RelE toxin. RelE is a part of a type II toxin-antitoxin module^55^ in which the toxin RelE acts as a ribosome-dependent endoribonuclease activated in diverse cellular processes, including nutrient starvation and persistence^56^. The antitoxin encoded by RelB counteracts RelE’s toxic activity by modifying the structure of the RelE catalytic domain^57^. During amino acid starvation, RelE inhibits translation by cleaving within the mRNA ribosomal A-site. Interestingly, our expression analysis conducted in NZM-poor medium showed that Srn_9344 slightly decreased, while RNA levels of Srn_9342, Srn_9345, and Srn_9346 increased. However, no RelB homologues could be identified next to Srn_9343 or elsewhere in the Newman genome, suggesting that Srn_9343 may not be a part of a type II toxin-antitoxin system in *S. aureus*. The architecture of the *srn_9342-9346* locus is unusual in *S. aureus*. The presence of an sRNA (Srn_9344) transcribed from the opposite strand of the peptide-coding Srn_9343, and at the same locus, suggests that Srn_9344 could function as a *cis*-antisense. We hypothesize that this pair may function as a type I toxin-antitoxin system. Apart from *S. aureus*, another unusual sRNA cluster was recently identified in the *Enterococcus faecalis* clinical isolate V583, where a chromosomal locus containing two toxin-antitoxin modules (type I and II) with *cis*-antisense transcription was discovered^58^.

Our search for ORFs within the 24 sRNAs mapped by RACE revealed that some of them may encode peptides or small proteins, although none of them are homologous to phenol-soluble modulins^45, 46^ or any peptide/proteins with a known function. Many published sRNAs were described as having coding capacities^11, 14, 21, 22^ and there are accumulating evidences for the existence of RNAs with dual functions in prokaryotes and eukaryotes^59, 60^. In *S. aureus*, this is the case for multifunctional regulatory RNAIII, that besides monitoring the expression of a large set of mRNA targets in its RNA form^2, 3^, encodes the delta-hemolysin. This raises the question of whether the term “noncoding RNA” sometimes used by the RNA community is still relevant and may suggest that some of the sRNAs described here may harbor dual-functions. Recent work in *S. aureus* showed that Srn_4340 (Teg23), which contains an ORF, is probably a small non-coding RNA rather than a small coding sequence, since no translation was seen at all^21^. In that study, the authors emphasized that current automatic annotation methods can lead to genome overannotation, therefore complicating the identification and study of new sRNA. It therefore appears that the distinction between the mRNA and sRNA world (apart from comparing RNA lengths) is probably more complicated and intertwined than expected, and that thorough experimental assessments of potential coding capacities and regulatory functions may be necessary.

The assignment of Srn identifiers as per a procedure established in SRD^22^ allowed us to investigate the distribution of the 24 *srns* within several *S. aureus* strains. This search revealed that seven of them were conserved among the strains we analyzed. Aligning these sRNA sequences revealed a high level of conservation, with only few SNPs. Such high conservation is usually observed for transcribed regions and is significantly decreased in true IGRs. One of the open questions is whether there are still additional sRNAs to discover in *S. aureus*. Based on the work we present here and on other recent studies^21^, all performed on different strains in the same clonal complex (CC 8), we can reasonably suppose that the annotation of the pan-sRNome is far from being complete. Therefore, the study of other clonal complexes and using various experimental conditions should help in the identification of novel sRNAs which could be specific to clonal complexes or conserved within the species and therefore belong to the sRNA core. Investigations could be extended to the study of strains deleted for transcription factors, since it was recently shown that sRNAs could belong to regulons in *S. aureus* ^30^. Indeed, studies of the expression of our novel sRNAs under various stress conditions revealed that they respond to few specific stresses, and that their expression is therefore tightly monitored. Interestingly, our data indicated that many of these novel sRNAs are downregulated under oxidative stress. This can be opposed to the observation made in N315 during the identification of the Teg sRNAs^11^, implying that each sRNA responds to its own set of physico-chemical modifications.

In conclusion, including the 24 novel sRNA coordinates in the SRD will enable subsequent RNA-Seq studies using various experimental conditions to monitor their physiological role. Global transcriptome approaches are necessary for an exhaustive characterization of the *S. aureus* RNome, and this step is essential before trying to understand its overarching role and function within the bacterium and especially during infection. Our work shows that the search for novel sRNAs in well-studied bacteria is valuable, and that this can be facilitated through setting conditions that mimic the *in vivo* environment, by using different RNA-Seq approaches, or by studying strains deleted for major transcription factors to start characterizing the complex connections between sRNAs and proteins in this major human pathogen.

## Methods

### Bacterial strains, growth, and stress conditions

The *S. aureus* strains used in this study are listed in Table 6. *S. aureus* was grown in Brain Heart Infusion broth (BHI, Oxoid) or in Tryptone Soya Broth (TSB, Oxoid) under agitation at 37 °C (except where stated). Growth was monitored by measuring the OD_600nm_ at different time points. For the analysis of RNA levels under different stresses, *S. aureus* was cultured in TSB broth up to an OD_600nm_ of 2 (exponential phase of growth). Stresses were induced by the addition of 10 mM H_2_O_2_ (oxidative stress), HCl (to lower the pH to 5.5), NaOH (to increase the pH to 9.5), or by temperature shifts to 18 °C or 42 °C (cold and heat shocks). In addition, 60 ml of culture was centrifuged at 4500 rpm for 8 min at room temperature and the pellet resuspended in TSB supplemented with 1 M NaCl (osmotic stress), NZM medium (stringent response), or fresh TSB as a control.

**Table 6:** Bacterial strains and plasmids used in this study.

Strains and plasmids	Characteristics	Reference	Accession number
***S. aureus*** **strains**			
Newman	Methicillin-sensible *S. aureus* strain	23	AP009351
N315	Methicillin-resistant *S. aureus* strain	62	BA000018
HG003	Derivative strain of NCTC8325	63	JPPU00000000
USA300 FPR3757	Community-acquired methicillin-resistant clone	38	CP000255
UAMS-1	Oxacillin-susceptible clinical isolate	64	JTK00000000
Newman-217	Newman Δ*rny*::*ermC*	35	
Newman-217 pCG296	Newman Δ*rny*::*ermC* with pCG296 for complementation	35	
Newman *Δsrn_3610_sprC*	Newman strain deleted for sRNA Srn_3610_SprC	25	
RN4220	Type I restriction-modification-deficient strain	65	
***E. coli*** **strain**	Srn_9343 under its promoter (100 nt) and with 3′ 3xFLAG	This study	
XL1 Blue			
**Plasmids**			
pYAR139			
pYAR140	Srn_9343 under its promoter (250 nt) and with 3′ 3xFLAG	This study	
pYAR144	Srn_9343 under its promoter (42 nt) and with 3′ 3xFLAG	This study	
pYAR146	Srn_9343 under its promoter (70 nt) and with 3′ 3xFLAG	This study	
pYAR148	Srn_9343 under P*veg* promoter and with 3′ 3xFLAG	This study	

### Total RNA extraction

Cells were harvested by centrifugation at 4500 rpm for 10 min and pellets washed with 500 µL of cold lysis buffer (20 mM sodium acetate, 1 mM EDTA, 0.5% SDS, pH 5.5). Cell pellets were broken using acid-washed glass beads (Sigma) in the presence of phenol (pH 4) in an FP120 FastPrep cell disruptor (MP Biomedicals) for 30 sec at a power setting of 6.5. Lysates were centrifuged at 16,000 g for 5 min at 4 °C. Total RNA were extracted with phenol/chloroform and precipitated overnight with sodium acetate.

### cDNA library construction and Illumina RNA sequencing

Overnight cultures of *S. aureus* were diluted in fresh BHI broth to an OD_600nm_ of 0.1 then cultured at 160 rpm for 5 hr at 37 °C. Total RNA were extracted as described above and treated with Amplification Grade DNase I (Invitrogen) to remove genomic contaminations. The absence of DNA was checked by qPCR in an Applied Biosystems 7500 instrument and RNA integrity verified on a Bioanalyzer (Agilent). Ribosomal RNAs were depleted using a Ribo-Zero Magnetic Kit (Epicentre) according to the manufacturer’s recommendations. Stranded cDNA libraries were prepared using the NEBNext Ultra Directional RNA Library Prep Kit for Illumina (New England Biolabs). The concentration, quality, and purity of the libraries were determined using a BioAnalyzer, a Qubit fluorometer (Invitrogen), and a Nanodrop spectrophotometer (Thermo Scientific). Libraries were sequenced on either an Illumina MiSeq instrument (single-end, 50 cycles) or an Illumina HiSeq 1500 system (rapid run, 200 cycles, paired-end), as per the manufacturer’s instructions.

### Reads mapping, analysis, and visualization

The Newman strain genome sequence and annotation file (in GFF format) was obtained from NCBI (ftp://ftp.ncbi.nlm.nih.gov/genomes/Bacteria/Staphylococcus_aureus_Newman_uid58839/). All of the SRD srns described for Newman strain^22^ were added to this GFF. Quality control of RNA-Seq reads and read mappings was performed as previously described^22^. SAM files were filtered on bitwise flag values^61^ to select properly paired reads. Fragments sorted in SAM files were counted by HTSeq count^31^ for stranded library with the union mode and differential expression calculated using DESeq^32^. BAM files were visualized using the Artemis browser^34^.

### Identification of novel RNA candidates by RNA-Seq

We used the DETR’PROK^27^ workflow of 43 steps to identify novel RNAs in *S. aureus* Newman. Briefly, the pipeline clustered overlapping RNA-Seq reads from BWA alignments to identify novel transcripts in intergenic regions using a GFF file that combined 2614 CDS; 56 tRNAs; 16 rRNAs; RNaseP RNA; 4.5 S RNA; tmRNA; and 504 SRD-listed *srns*. We set DETR’PROK to identify and retain clusters of more than 12 overlapping reads that were located at least 25 nt apart from existing annotations and had a length over 50 nt. DETR’PROK then produced a list of candidates in a GFF annotation file.

### Northern blot analysis and RACE-mapping

Northern blots were performed as previously described^48^. Briefly, 15 µg of total RNA were loaded and separated in 7% polyacrylamide/8 M urea gels. To estimate the actual length of transcripts, a DynaMarker® Prestain Marker for Small RNA Plus was loaded into each gel. RNAs were probed with ^γ32^P 5′ endlabeled oligonucleotides (File S4) and detected using a Typhoon FLA 9500 scanner (GE Healthcare). RACE-mapping was performed as previously described^41^ with some modifications (see below), using the primers listed in File S4. To distinguish the transcription start sites from RNA maturation of transcripts, the RNA were treated with Terminator 5′-Phosphate-Dependent Exonuclease (Epicentre) to degrade 5′monophosphate RNAs, then with polyphosphatase (Epicentre) to remove pyrophosphate from TSS RNA. Primary transcript enriched-RNAs were then ligated as previously described^41^.

### Peptide cloning and western blots

*srn_9343* was cloned in pCN35 under its own promoter or under P*veg* promoter^44^ and in fusion with a 3xFLAG (GACTACAAGGACCACGACGGTGACTACAAGGACCAC GACATCGACTACAAGGACGACGACGACAAGTGA) using the primers listed in File S4. Recombinant plasmids (Table 6) were transformed in *S. aureus* as previously described^30^. *S. aureus* was cultured in BHI broth at 37 °C and under agitation until reaching an OD_600nm_ of 6. Cells were harvested by centrifugation, and cellular protein extracts were prepared with protease inhibitors as previously described^41^. Proteins from the supernatant were precipitated with TCA and then with acetone as previously described^41^. For western blots, samples were separated on 16% Tricine-SDS-PAGE and transferred onto Hybond-P PVDF membranes (Amersham). After overnight blocking at 4 °C, membranes were incubated with ANTI-FLAG horseradish peroxidase-conjugated antibodies (HRP) (Sigma). The membranes were revealed using an ECL Plus Western Blotting Detection Kit (Amersham) and scanned with an ImageQuant LAS 4000 imager.

### RT-qPCR

Total RNA extraction was performed as described above^42^. RNA samples (1 µg of total RNA) were treated with 1 unit of Amplification Grade DNase I, and the absence of DNA contamination was checked by qPCR using an Applied Biosystems 7500 system. cDNA were synthesized from total RNA (250 ng) using a High-Capacity cDNA Archive Kit (Applied Biosystems) and specific primers to discriminate the strand transcribed. Real-time PCR was done using a QuantiTect SYBR Green PCR Kit (Qiagen) in an Applied Biosystems 7500 real-time PCR system. Data were analyzed using the comparative critical threshold (ΔΔCT) method as previously described: the target RNA amounts were compared with HU or 5 S RNA, which served as internal controls^25^. The primers used for cDNA synthesis and qPCR are listed in File S4.

### Data Deposition

The RNA-Seq data generated during this study (by MiSeq and Hiseq) was submitted to the GEO repository under accession number GSE89487. Additionally, reads obtained from the work of Sassi *et al*.^22^, previously registered with GEO under accession number GSE64026 were used to enhance the number of replicates.

## Electronic supplementary material

Supplementary information

Dataset 1

Dataset 2

Dataset 3

Dataset 4
