# Supplementary material for: sRNA and cis-antisense sRNA identification in Staphylococcus aureus highlights an unusual sRNA gene cluster with one encoding a secreted peptide
Source: Sci Rep. 2017 Jul 4;7:4565. doi: 10.1038/s41598-017-04786-3 (PMC5496865; doi:10.1038/s41598-017-04786-3)
Supplement: Supplementary file 1 — Supplementary information [file 41598_2017_4786_MOESM1_ESM.pdf]

sRNA and *cis*-antisense sRNA identification in *Staphylococcus aureus* highlights an unusual sRNA gene cluster with one encoding a secreted peptide

Julie Bronsard, Gaetan Pascreau, Mohamed Sassi, Tony Mauro, Yoann Augagneur*, and Brice Felden
Inserm U1230 Biochimie Pharmaceutique, Université de Rennes 1, Rennes, France

*Address correspondence to Yoann Augagneur, yoann.augagneur@univ-rennes1.fr (or alternatively to brice.felden@univ-rennes1.fr).

Supplementary Information

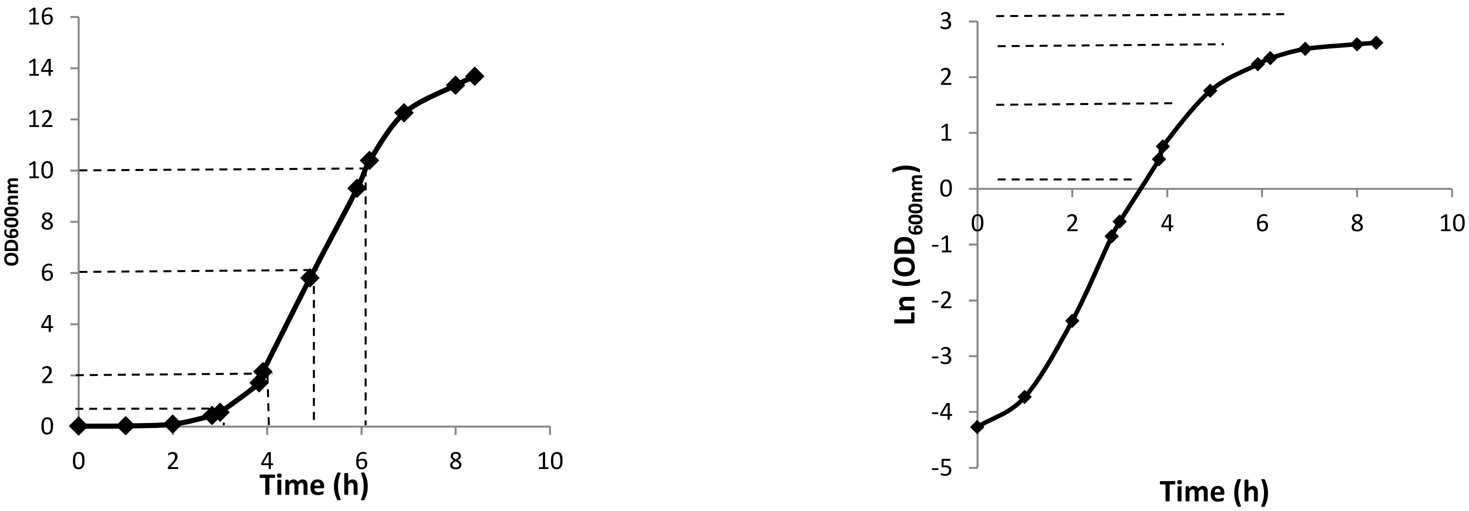

Figure S1: Growth curve of Staphylococcus aureus Newman in BHI broth. Cells were inoculated at an OD600nm of 0.02 and cultured at 37°C under agitation at 160 rpm. The dotted lines correspond to the time in hours (h) when cells were harvested prior RNA extraction.

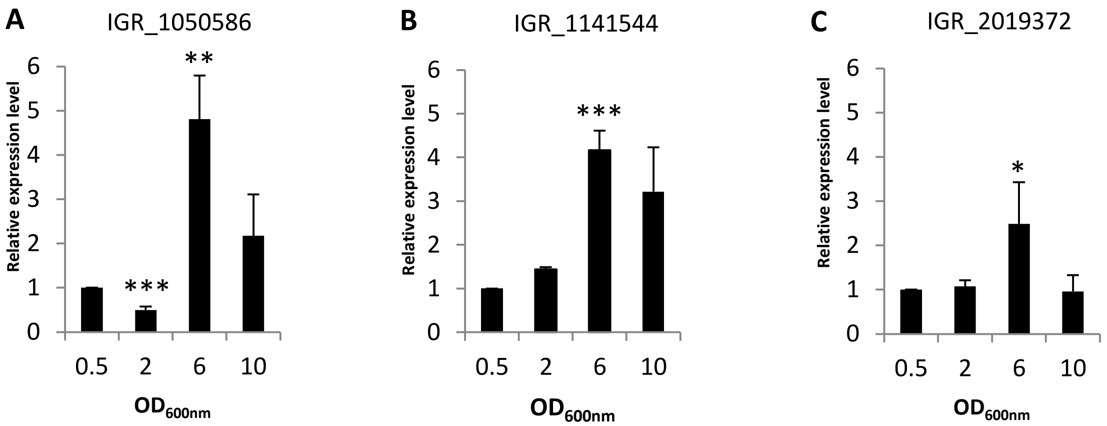

Figure S2: Relative RNA levels of (A) IGR_1050586, (B) IGR_1141544, and (C) IGR_2019372 in *Staphylococcus aureus* Newman based on northern blots. Cells were grown in BHI medium and harvested at 0.5, 2, 6, and 10 OD_{600nm}. tmRNA was used as an internal loading control, and the relative expression level determined at an OD_{600nm} of 0.5 was set to one for each gene tested. Data presented are the means of three independent experiments. A student t-test was done to determine significant differences with the condition at OD_{600nm} of 0.5.

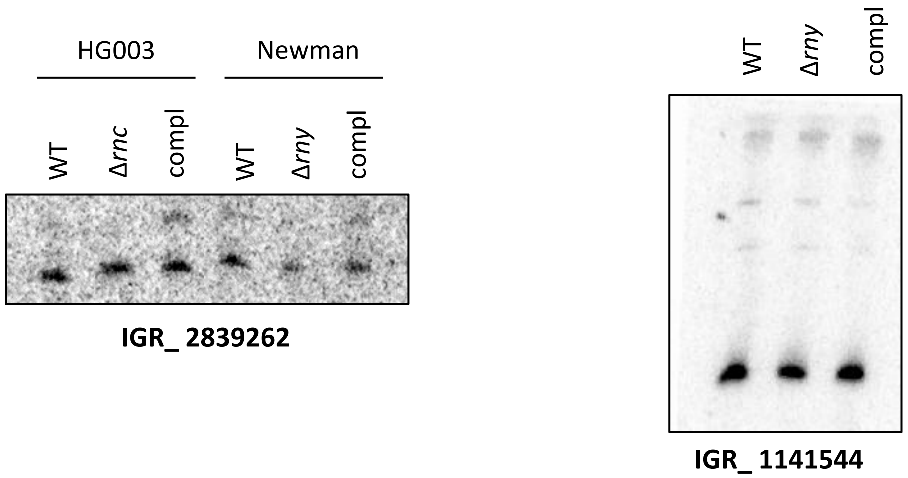

Figure S3 : Expression profile of IGR_2839262 and IGR_1141544 RNase-deficient strains. Northern blot were performed using probes specific to the targets on RNAs extracted from HG003 (*Δrnc,* RNaseIII mutant) or Newman (*Δrny*: RNaseY mutant) cultured in BHI medium. The expression profile of IGR_2839262 was monitored in HG003 *ΔrnaseIII* and in Newman *ΔrnaseY* mutants of *S. aureus,* while IGR_1141544 expression was only monitored in Newman *ΔrnaseY* since the targeted region is absent in HG003.WT, wild-type; compl, complemented strain.

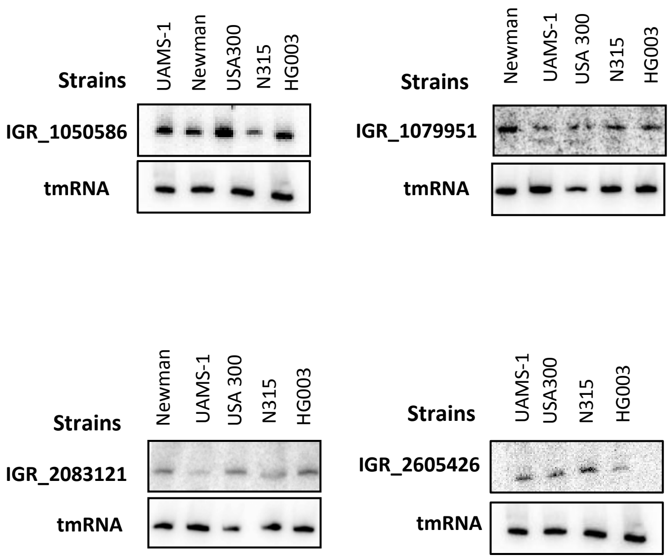

Figure S4 : IGR 1050586, 1079951, 2083121, and 2605426 expression in five *Staphylococcus aureus* strains. Northern blots were performed using target-specific probes on RNA extracted from UAMS-1, Newman, USA300, N315, and HG003 cultured in BHI medium until OD_{600nm} = 6. tmRNA was used as an internal loading control.

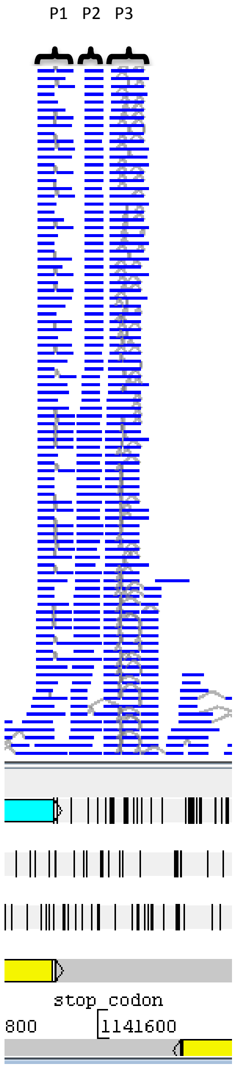

Probe	5'-end	3'-end	Length
P1	1141253	1141502	249
P2	1141525	1141656	131
P3	1141676	1141990	314

Figure S5: Artemis visualization of paired-end reads mapped onto the IGR_1141544 locus.

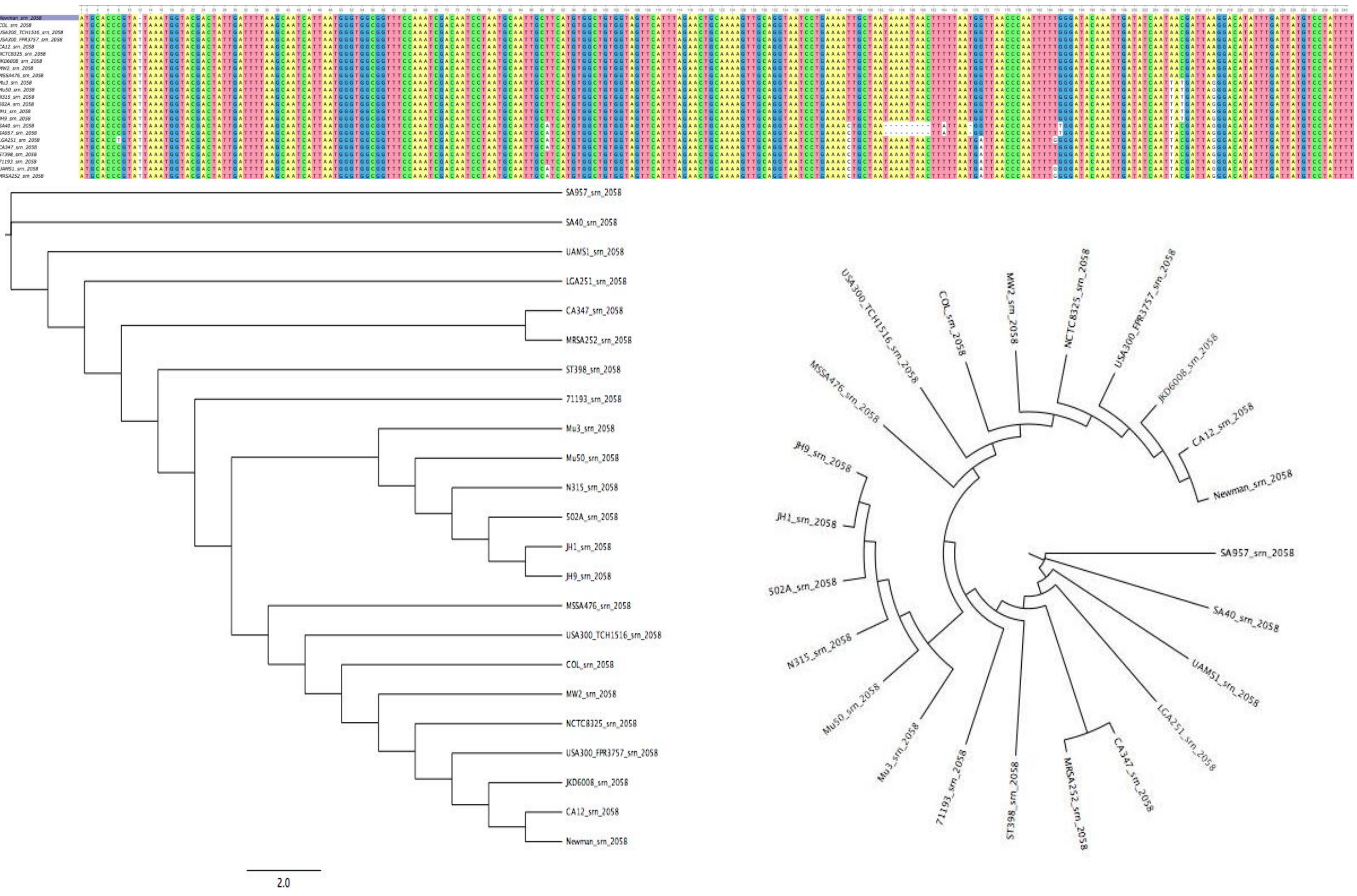

Figure S6: Alignment of the retained new sRNAs in a set of *S. aureus* strains.

srn_2058

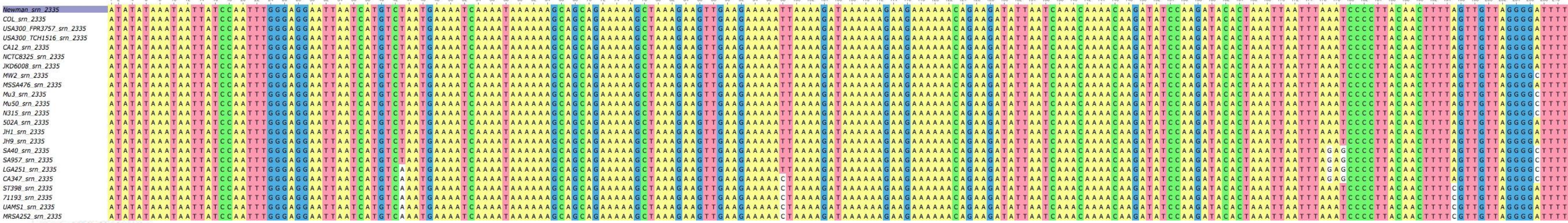

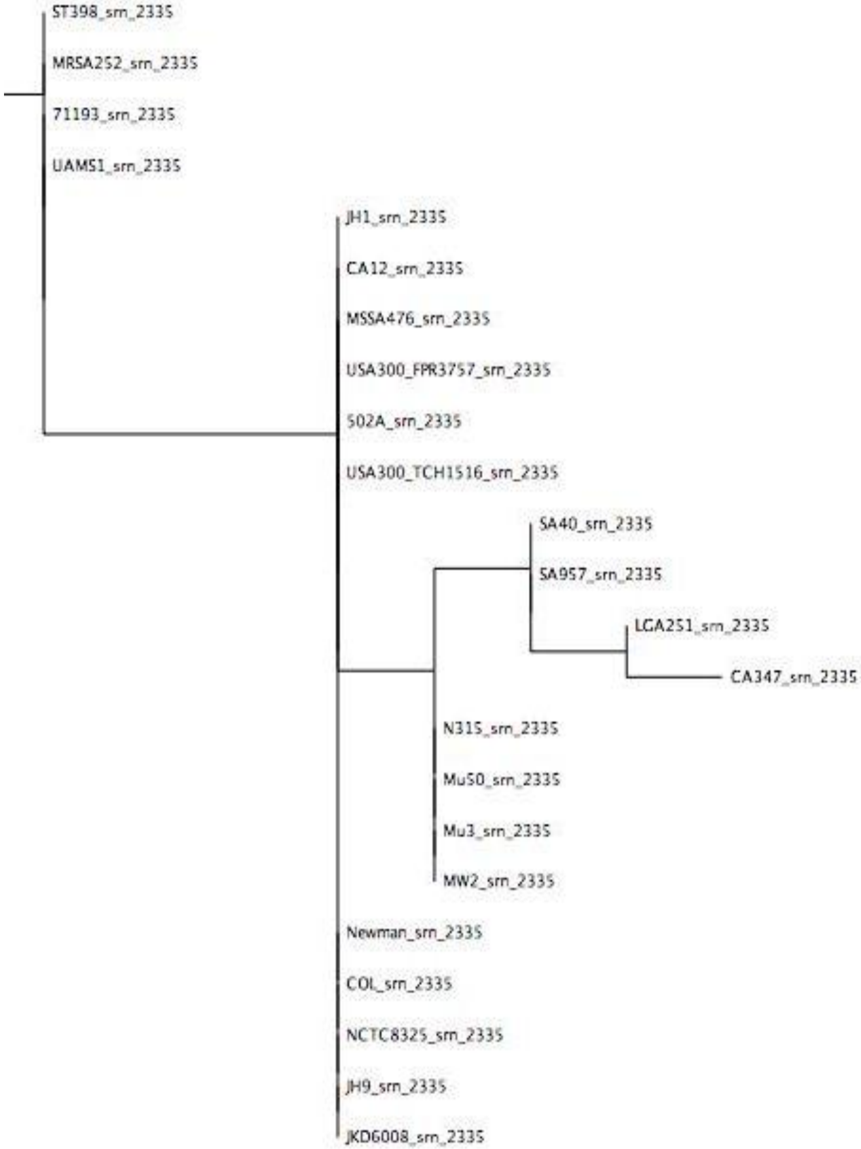

ST398_srn_2335
MRSA252_srn_2335
71193_srn_2335
UAMS1_srn_2335
JH1_srn_2335
CA12_srn_2335
MSSA476_srn_2335
USA300_FPR3757_srn_2335
502A_srn_2335
USA300_TCH1516_srn_2335
SA40_srn_2335
SA957_srn_2335
LGA251_srn_2335
CA347_srn_2335
N315_srn_2335
Mu50_srn_2335
Mu3_srn_2335
MW2_srn_2335
Newman_srn_2335
COL_srn_2335
NCTC8325_srn_2335
JH9_srn_2335
JKD6008_srn_2335

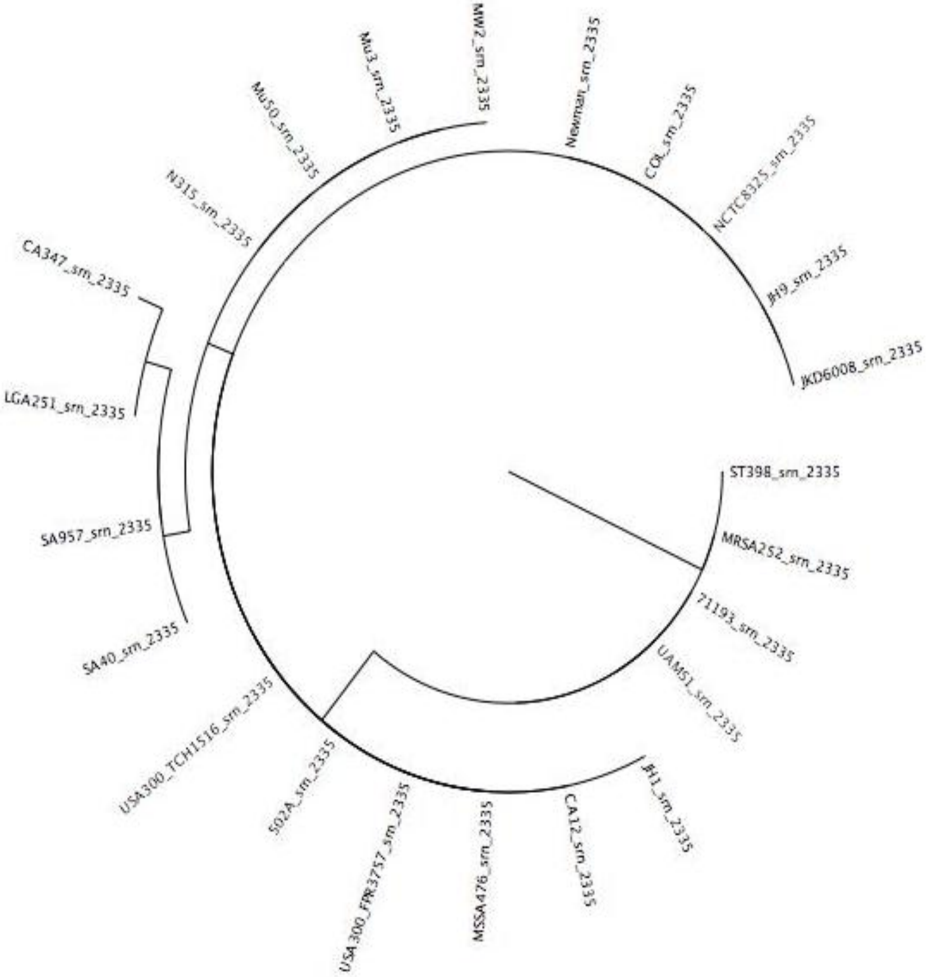

MW2_srn_2335
Mu3_srn_2335
Mu50_srn_2335
Newman_srn_2335
COL_srn_2335
NCTC8325_srn_2335
N315_srn_2335
CA347_srn_2335
JH9_srn_2335
JKD6008_srn_2335
LGA251_srn_2335
ST398_srn_2335
SA957_srn_2335
MRSA252_srn_2335
71193_srn_2335
SA40_srn_2335
UAMS1_srn_2335
USA300_TCH1516_srn_2335
502A_srn_2335
USA300_FPR3757_srn_2335
MSSA476_srn_2335
CA12_srn_2335
JH1_srn_2335

srn_2335

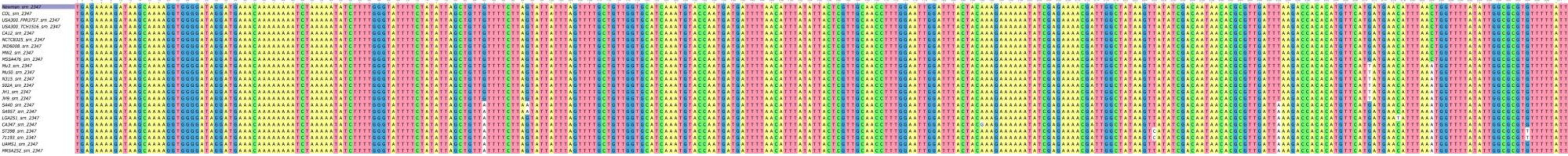

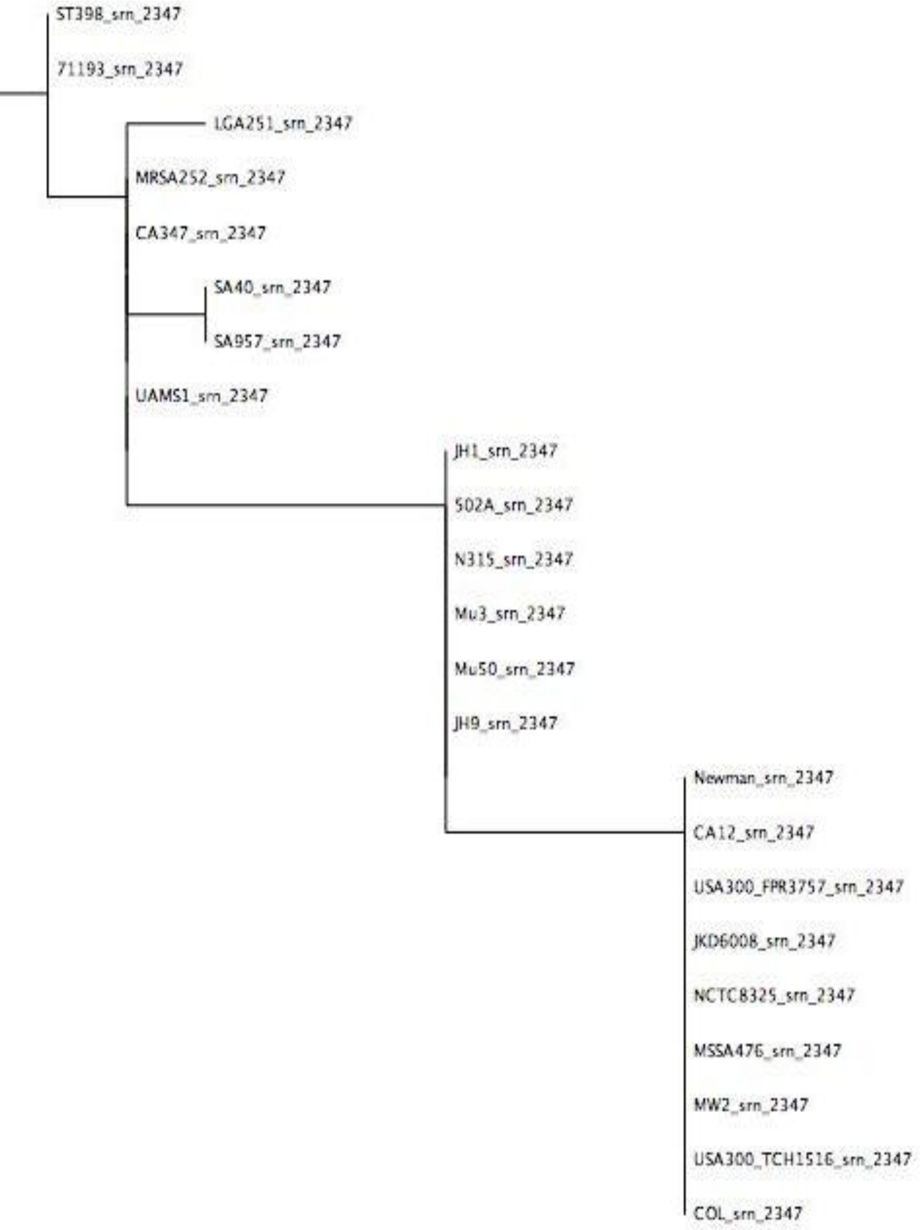
ST398_srn_2347
71193_srn_2347
LGA251_srn_2347
MRSA252_srn_2347
CA347_srn_2347
SA40_srn_2347
SA957_srn_2347
UAMS1_srn_2347
JH1_srn_2347
502A_srn_2347
N315_srn_2347
Mu3_srn_2347
Mu50_srn_2347
JH9_srn_2347
Newman_srn_2347
CA12_srn_2347
USA300_FPR3757_srn_2347
JKD6008_srn_2347
NCTC8325_srn_2347
MSSA476_srn_2347
MW2_srn_2347
USA300_TCH1516_srn_2347
COL_srn_2347

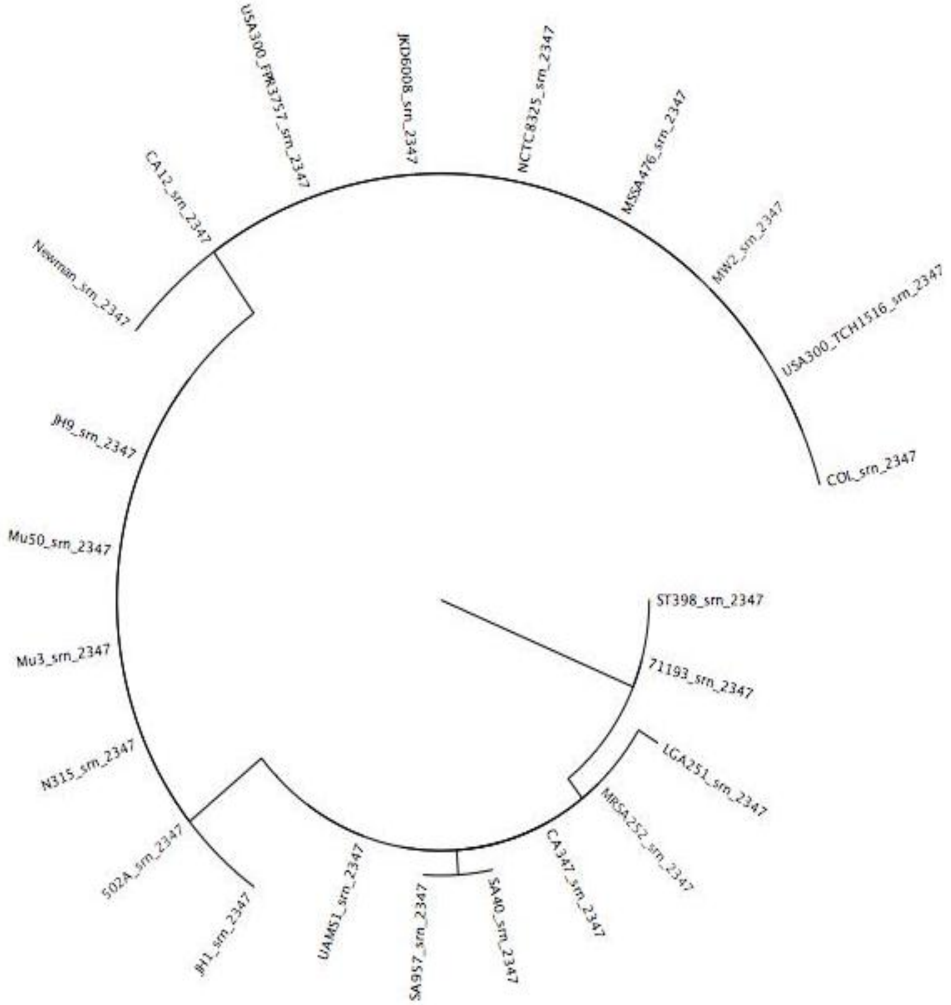
USA300_FPR3757_srn_2347
JKD6008_srn_2347
NCTC8325_srn_2347
MSSA476_srn_2347
MW2_srn_2347
USA300_TCH1516_srn_2347
COL_srn_2347
CA12_srn_2347
Newman_srn_2347
JH9_srn_2347
Mu50_srn_2347
Mu3_srn_2347
N315_srn_2347
502A_srn_2347
JH1_srn_2347
UAMS1_srn_2347
SA957_srn_2347
SA40_srn_2347
CA347_srn_2347
MRSA252_srn_2347
LGA251_srn_2347
71193_srn_2347
ST398_srn_2347

srn_2347

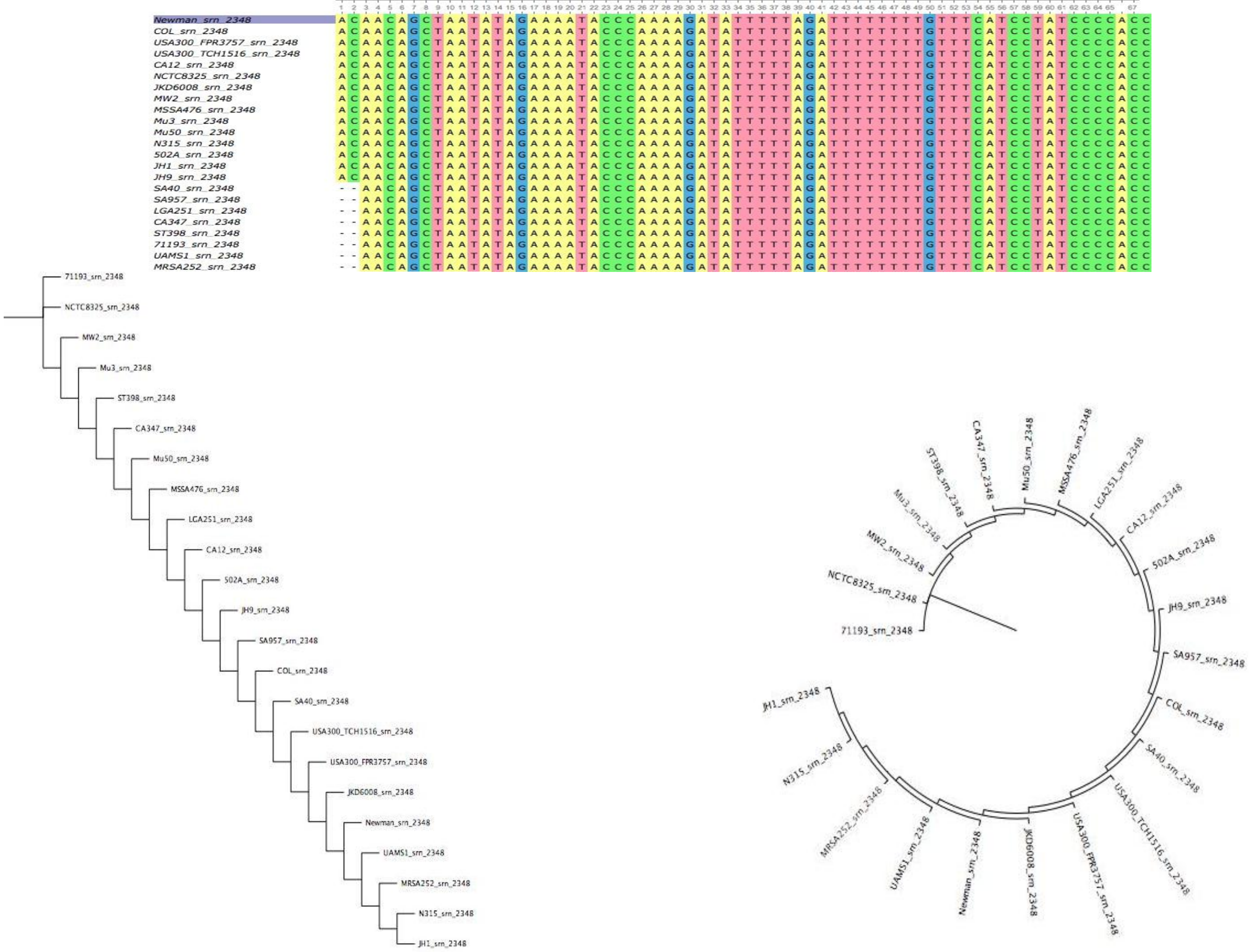

Newman_srn_2348
COL_srn_2348
USA300_FPR3757_srn_2348
USA300_TCH1516_srn_2348
CA12_srn_2348
NCTC8325_srn_2348
JKD6008_srn_2348
MW2_srn_2348
MSSA476_srn_2348
Mu3_srn_2348
Mu50_srn_2348
N315_srn_2348
502A_srn_2348
JH1_srn_2348
JH9_srn_2348
SA40_srn_2348
SA957_srn_2348
LGA251_srn_2348
CA347_srn_2348
ST398_srn_2348
71193_srn_2348
UAMS1_srn_2348
MRSA252_srn_2348

srn_2348

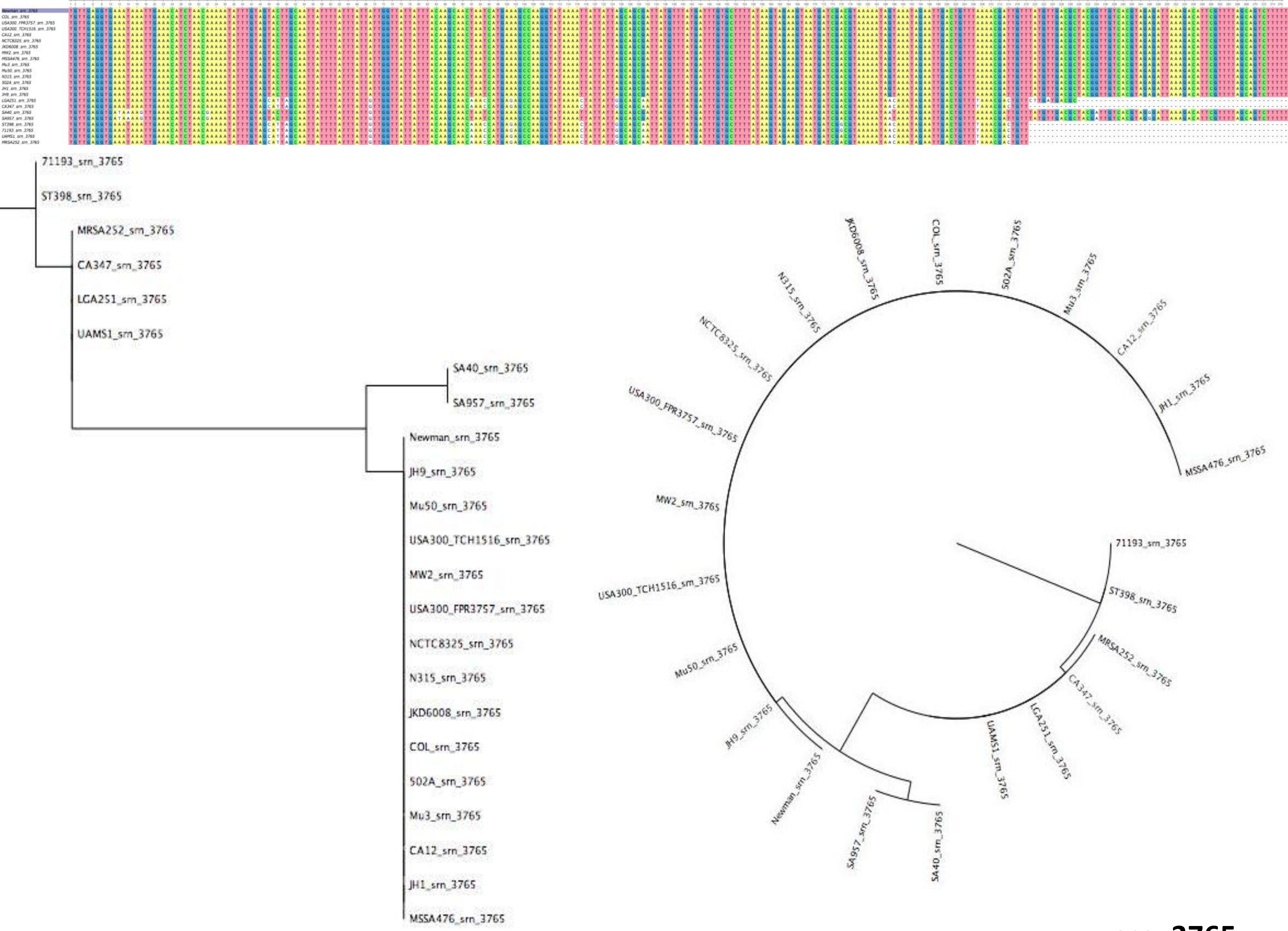
71193_srn_3765
ST398_srn_3765
MRSA252_srn_3765
CA347_srn_3765
LGA251_srn_3765
UAMS1_srn_3765
SA40_srn_3765
SA957_srn_3765
Newman_srn_3765
JH9_srn_3765
Mu50_srn_3765
USA300_TCH1516_srn_3765
MW2_srn_3765
USA300_FPR3757_srn_3765
NCTC8325_srn_3765
N315_srn_3765
JKD6008_srn_3765
COL_srn_3765
502A_srn_3765
Mu3_srn_3765
CA12_srn_3765
JH1_srn_3765
MSSA476_srn_3765

srn_3765

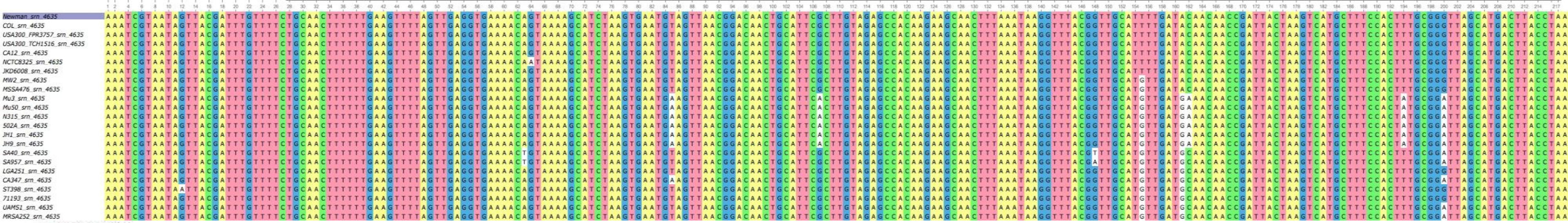

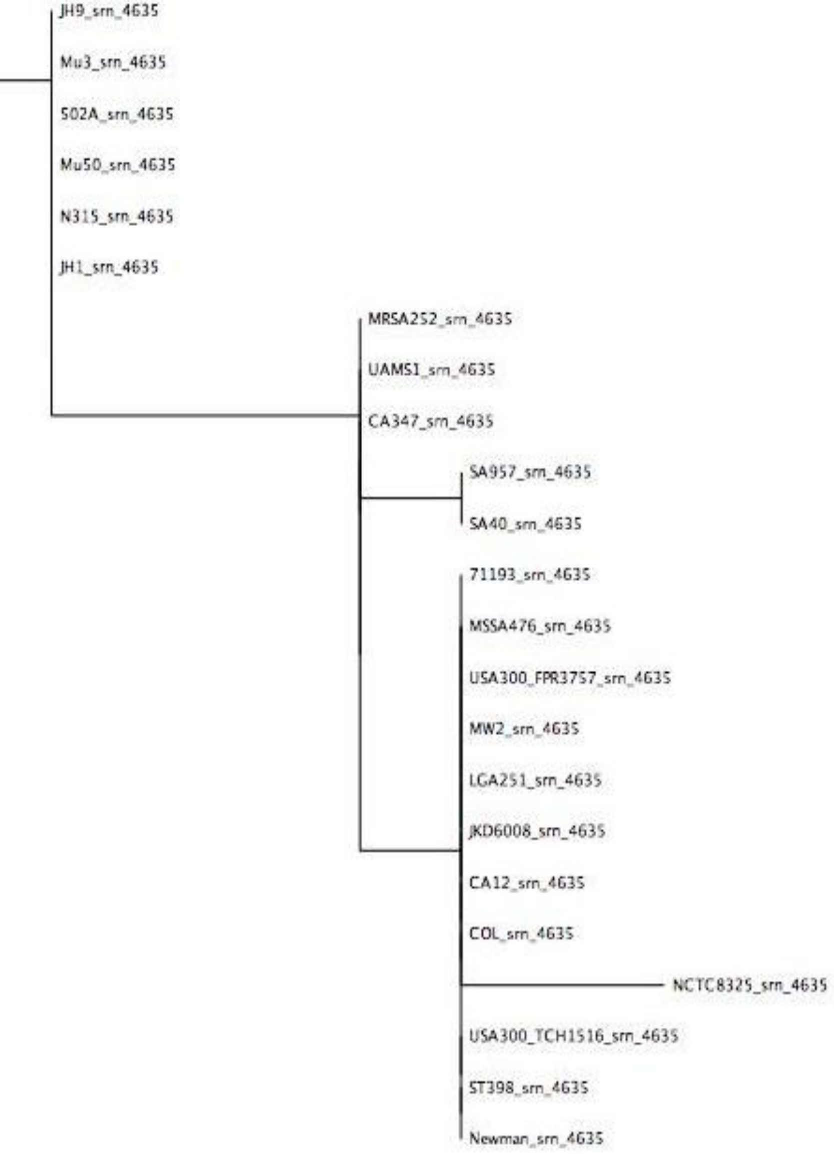
JH9_srn_4635
Mu3_srn_4635
502A_srn_4635
Mu50_srn_4635
N315_srn_4635
JH1_srn_4635
MRSA252_srn_4635
UAMS1_srn_4635
CA347_srn_4635
SA957_srn_4635
SA40_srn_4635
71193_srn_4635
MSSA476_srn_4635
USA300_FPR3757_srn_4635
MW2_srn_4635
LGA251_srn_4635
JKD6008_srn_4635
CA12_srn_4635
COL_srn_4635
NCTC8325_srn_4635
USA300_TCH1516_srn_4635
ST398_srn_4635
Newman_srn_4635

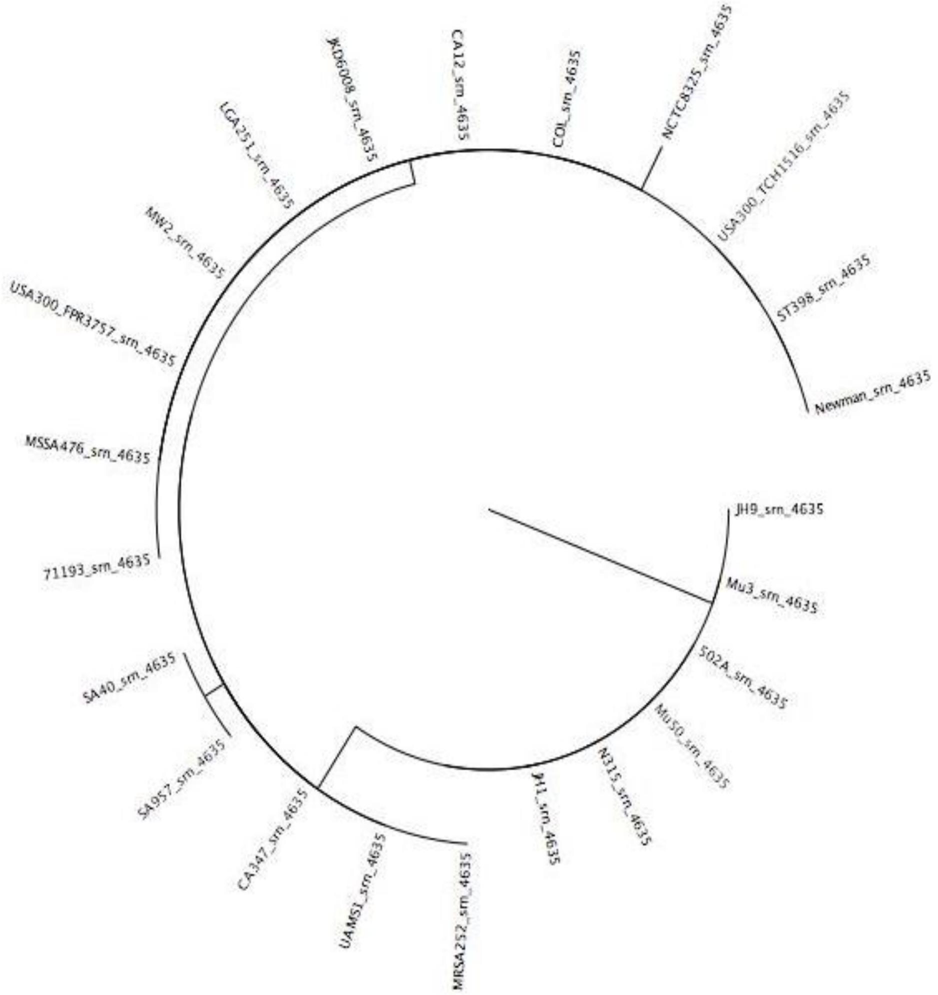
CA12_srn_4635
COL_srn_4635
NCTC8325_srn_4635
USA300_TCH1516_srn_4635
ST398_srn_4635
Newman_srn_4635
JKD6008_srn_4635
LGA251_srn_4635
MW2_srn_4635
USA300_FPR3757_srn_4635
MSSA476_srn_4635
71193_srn_4635
JH9_srn_4635
Mu3_srn_4635
502A_srn_4635
Mu50_srn_4635
N315_srn_4635
JH1_srn_4635
SA40_srn_4635
SA957_srn_4635
CA347_srn_4635
UAMS1_srn_4635
MRSA252_srn_4635

srn_4635

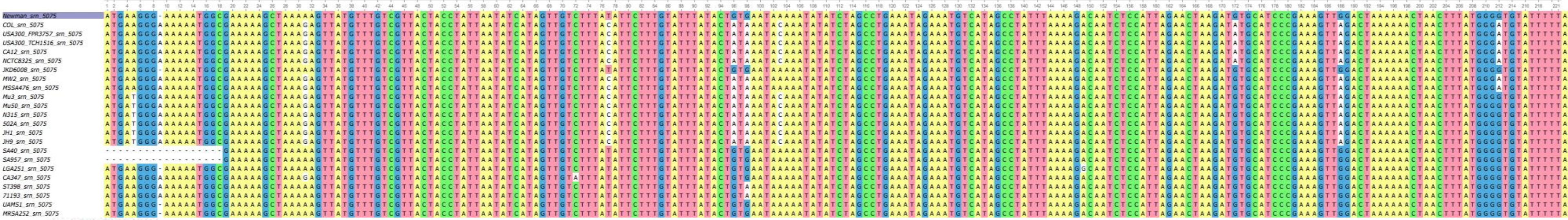

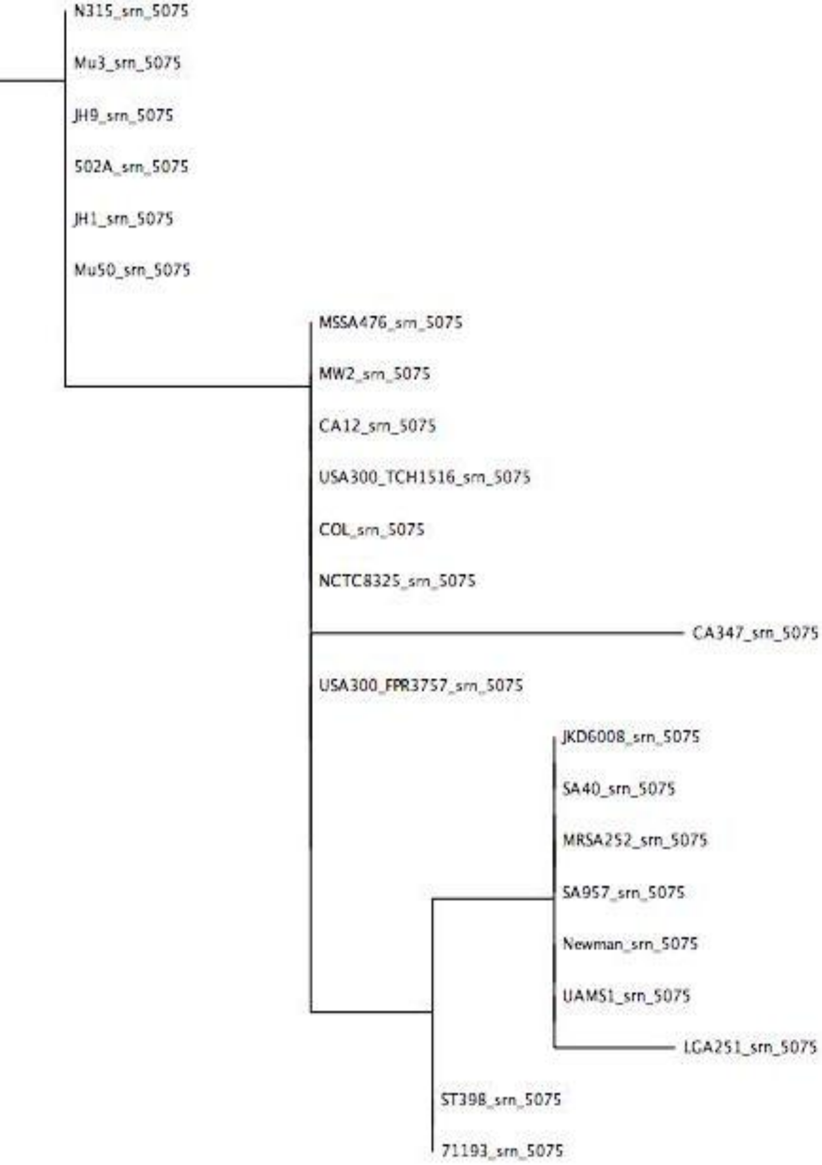
N315_srn_5075
Mu3_srn_5075
JH9_srn_5075
502A_srn_5075
JH1_srn_5075
Mu50_srn_5075
MSSA476_srn_5075
MW2_srn_5075
CA12_srn_5075
USA300_TCH1516_srn_5075
COL_srn_5075
NCTC8325_srn_5075
CA347_srn_5075
USA300_FPR3757_srn_5075
JKD6008_srn_5075
SA40_srn_5075
MRSA252_srn_5075
SA957_srn_5075
Newman_srn_5075
UAMS1_srn_5075
LGA251_srn_5075
ST398_srn_5075
71193_srn_5075

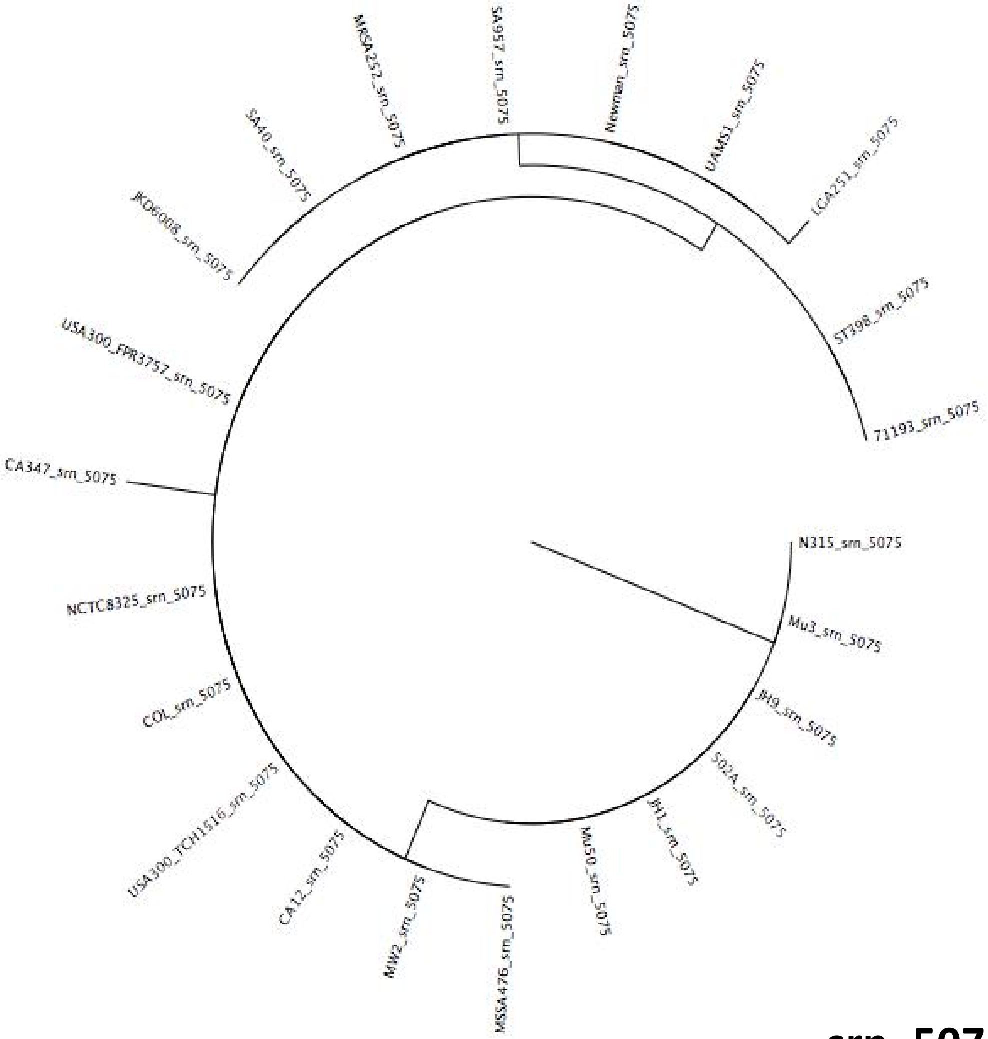
SA957_srn_5075
MRSA252_srn_5075
Newman_srn_5075
UAMS1_srn_5075
SA40_srn_5075
LGA251_srn_5075
JKD6008_srn_5075
ST398_srn_5075
USA300_FPR3757_srn_5075
71193_srn_5075
CA347_srn_5075
N315_srn_5075
NCTC8325_srn_5075
Mu3_srn_5075
COL_srn_5075
JH9_srn_5075
502A_srn_5075
USA300_TCH1516_srn_5075
JH1_srn_5075
CA12_srn_5075
Mu50_srn_5075
MW2_srn_5075
MSSA476_srn_5075

srn_5075

A- Cellular extract

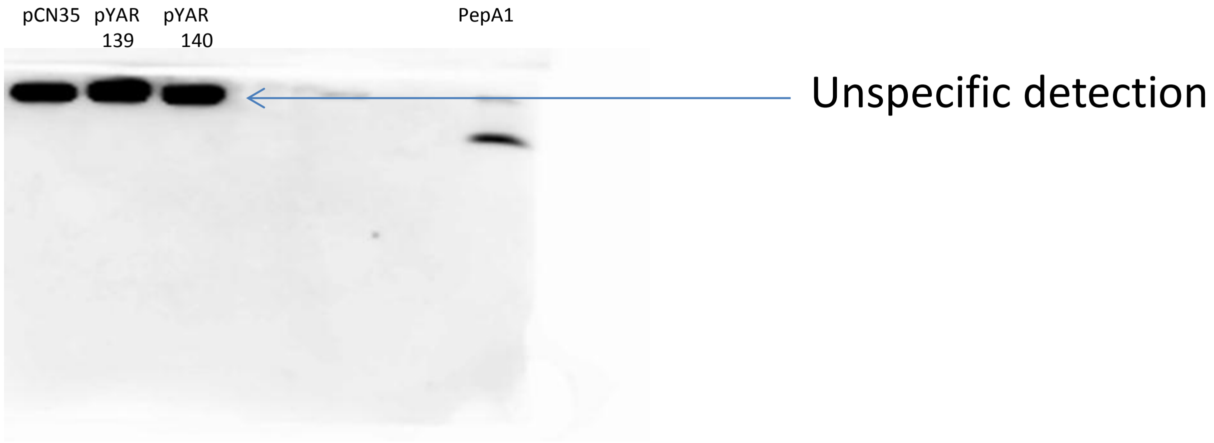

B- Extracellular proteins

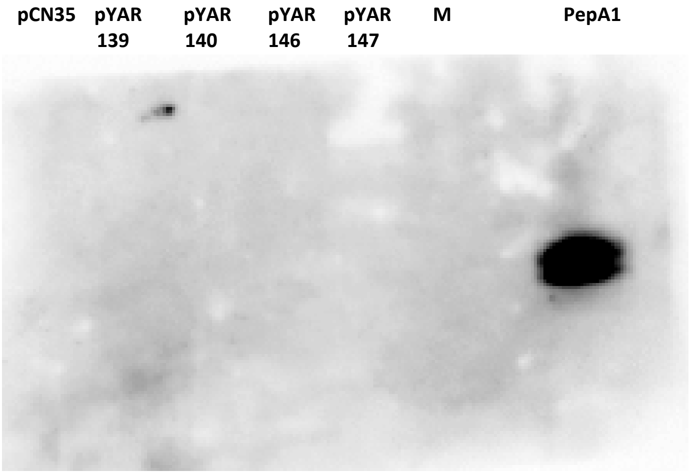

Figure S7: Western blots performed on cell extracts or secreted proteins of Newman strain transformed with appropriate plasmids.
